# Are lower levels of red blood cell transfusion more cost-effective than liberal levels after cardiac surgery? Findings from the TITRe2 randomised controlled trial

**DOI:** 10.1136/bmjopen-2016-011311

**Published:** 2016-08-01

**Authors:** E A Stokes, S Wordsworth, D Bargo, K Pike, C A Rogers, R C M Brierley, G D Angelini, G J Murphy, B C Reeves

**Affiliations:** 1 Nuffield Department of Population Health, Health Economics Research Centre, University of Oxford, Oxford, UK; 2 Eli Lilly and Company Limited, Lilly House, Basingstoke, Hampshire, UK; 3 Clinical Trials and Evaluation Unit, School of Clinical Sciences, University of Bristol, Bristol, UK; 4 Bristol Heart Institute, School of Clinical Sciences, University of Bristol, Bristol, UK; 5 Department of Cardiovascular Sciences and NIHR Leicester Biomedical Research Unit in Cardiovascular Medicine, University of Leicester, Leicester, UK

**Keywords:** blood transfusion, cost-effectiveness

## Abstract

**Objective:**

To assess the incremental cost and cost-effectiveness of a restrictive versus a liberal red blood cell transfusion threshold after cardiac surgery.

**Design:**

A within-trial cost-effectiveness analysis with a 3-month time horizon, based on a multicentre superiority randomised controlled trial from the perspective of the National Health Service (NHS) and personal social services in the UK.

**Setting:**

17 specialist cardiac surgery centres in UK NHS hospitals.

**Participants:**

2003 patients aged >16 years undergoing non-emergency cardiac surgery with a postoperative haemoglobin of <9 g/dL.

**Interventions:**

Restrictive (transfuse if haemoglobin <7.5 g/dL) or liberal (transfuse if haemoglobin <9 g/dL) threshold during hospitalisation after surgery.

**Main outcome measures:**

Health-related quality of life measured using the EQ-5D-3L to calculate quality-adjusted life years (QALYs).

**Results:**

The total costs from surgery up to 3 months were £17 945 and £18 127 in the restrictive and liberal groups (mean difference is −£182, 95% CI −£1108 to £744). The cost difference was largely attributable to the difference in the cost of red blood cells. Mean QALYs to 3 months were 0.18 in both groups (restrictive minus liberal difference is 0.0004, 95% CI −0.0037 to 0.0045). The point estimate for the base-case cost-effectiveness analysis suggested that the restrictive group was slightly more effective and slightly less costly than the liberal group and, therefore, cost-effective. However, there is great uncertainty around these results partly due to the negligible differences in QALYs gained.

**Conclusions:**

We conclude that there is no clear difference in the cost-effectiveness of restrictive and liberal thresholds for red blood cell transfusion after cardiac surgery.

**Trial registration number:**

ISRCTN70923932; Results.

**Strengths and limitations of this study**

- The cost-effectiveness analysis was based on data collected for a large randomised controlled trial, the Transfusion Indication Threshold Reduction trial, which randomised four times more participants than the next largest trial comparing restrictive and liberal transfusion thresholds after cardiac surgery.
- Very detailed individual patient cost and quality-of-life data were collected from the trial which achieved a high level of completeness of follow-up.
- Only participants who breached the liberal threshold were randomised; this avoided diluting the treatment effect with similar numbers of participants in each group who were unlikely to be transfused.
- The unit costs of administering red blood cells used in this study captured the nursing costs associated with transfusion, but not other inputs such as those of the transfusion laboratory.

## Introduction

Perioperative anaemia is common after cardiac surgery and is associated with adverse clinical outcomes, such as stroke, acute kidney injury and death.^1–3^ Transfusion of allogeneic red blood cells is a mainstay treatment for acute anaemia and, on average, over 50% of adult cardiac surgery patients receive a perioperative blood transfusion.^4^ ^5^ Cardiac surgery consumes a substantial proportion of blood supplies; over 6% of all red blood cell usage in the UK occurs in cardiac surgery.^6^

Red blood cell transfusion is essential in some cardiac surgical patients for the management of life-threatening haemorrhage. In most cases, however, decisions to transfuse are made because the haemoglobin (Hb) concentration has fallen to a level or threshold at which the surgeon or critical care anaesthetist responsible for a patient's care feels uncomfortable.^2^ ^7^ ^8^ The transfusion threshold varies between different cardiac surgery units across the UK and between different doctors, which contributes to the wide variation in blood usage observed in cardiac surgical units (25–95%).^4^ ^5^ ^9^ A key reason for the threshold variation is the lack of evidence regarding what constitutes a ‘safe’ level of anaemia following cardiac surgery.

The Transfusion Indication Threshold Reduction (TITRe2) trial was designed to test the hypothesis that a restrictive threshold for red blood cell transfusion (Hb <7.5 g/dL) after cardiac surgery would reduce postoperative morbidity and health service costs compared to a liberal threshold (Hb <9 g/dL). The primary clinical outcome for TITRe2 was a composite of a serious infectious or ischaemic event within 3 months of randomisation. The trial results reported no difference in the primary clinical outcome between the transfusion groups (the primary outcome was observed in 35.1% and 33.0% of participants in the restrictive and liberal groups, respectively). This finding supports the use of either transfusion threshold as evaluated in the trial. This might suggest a restrictive threshold is preferred since this reduces unnecessary transfusions. However, analyses of a secondary outcome, mortality at 90 days, found a borderline significant difference between the groups (4.2% of patients in the restrictive group died vs 2.6% of patients in the liberal group, p=0.045) creating uncertainty about recommending restrictive transfusion after cardiac surgery.

Given the pressures on healthcare resources and the costs and scarcity of red blood cells, it is important to consider the cost-effectiveness as well as the clinical effectiveness of alternative transfusion thresholds. We are unaware of any previous trial-based economic evaluation assessing the cost-effectiveness of alternative transfusion thresholds after cardiac surgery. This paper reports the methods and results of the within-trial cost-effectiveness analysis for the TITRe2 trial.

## Methods

Our economic evaluation was based on the TITRe2 trial (ISRCTN70923932) which provided highly detailed data on resource use and health-related quality of life (HRQoL) of participants. The trial methods and results are reported in detail elsewhere,^7^ ^10^ and the trial CONSORT diagram is provided in the online supplementary appendix 1 of this paper. In summary, TITRe2 was a multicentre superiority trial in which patients aged >16 years having non-emergency cardiac surgery, whose Hb dropped to <9 g/dL during the postoperative hospital stay, were randomised to a restrictive threshold (transfuse if Hb <7.5 g/dL) or a liberal threshold (transfuse if Hb<9 g/dL). Patients were recruited from 17 specialist cardiac surgery centres in UK National Health Service (NHS) hospitals between July 2009 and February 2013.

10.1136/bmjopen-2016-011311.supp1 Supplementary appendices

Our analysis was conducted from an NHS and personal social services perspective, as recommended by the UK National Institute for Health and Care Excellence.^11^ The economic evaluation comprised a within-trial cost-effectiveness analysis, with the main outcome measure being quality-adjusted life years (QALYs), and took a 3-month time horizon, as we anticipated that most major resource use would occur within 3 months of cardiac surgery. Surgery was chosen as the time origin for our analyses (rather than the point of randomisation, as was the case with the analysis of effectiveness), in order to capture the resources that would be required for the intervention from a decision-maker's perspective, that is, to include all relevant costs (and effects) involved in delivering the cardiac surgery.

### Resource use and costs

Resource use data were collected on all significant health service resource inputs for the trial participants up to the point of the 3 month follow-up. During the index hospital admission, data collection was integrated into the trial case report forms; data were collected on blood products transfused, inpatient days by ward type, type of cardiac surgery and reoperations, medications and complications. At 3 months postoperatively, a bespoke resource use questionnaire was used to obtain estimates of healthcare resources used since hospital discharge, for example, readmissions to hospital and further contact with health professionals in primary or secondary care. The costs of unrelated care postdischarge were excluded. For example, our analysis included the cost of readmissions for hypertension and angina, but excluded the cost of readmissions for treatment of cancer.

Unit costs used to value hospital and community healthcare resource use were largely obtained from national sources, for example, NHS Blood and Transplant (NHSBT) price lists for blood products, the National Schedule of Reference Costs for intensive care, high-dependency and cardiac ward costs, MRI and CT scans and many complications, and Unit Costs of Health and Social Care for community costs.^12–14^ All unit costs are provided in the online supplementary appendix 2. Resources were valued in 2012/2013 pounds sterling; any unit costs in pre-2012/2013 prices were inflated to 2012/2013 using the Hospital and Community Health Services inflation index.^14^ Costs of drugs given in hospital were taken from the Electronic Marketing Information Tool where possible, which provides the reduced prices paid for generic drugs in hospital.^15^ Drug costs not available from this source or prescribed in the community were taken from the British National Formulary.^16^

### HRQoL and QALYs

The main outcome measure for the economic evaluation was HRQoL, using QALYs, which were derived from EQ-5D-3L utilities (measured on a continuous scale and time under observation). The EQ-5D was administered to participants preoperatively, and at 6 weeks and 3 months postoperatively. The analysis of QALYs required baseline utility to be modelled as a covariate; the correlation between baseline and 3-month EQ-5D-3L utilities was assumed to be ≥0.3. Respondents were assigned valuations derived from published UK population tariffs.^17^ The number of QALYs accrued by each participant was calculated assuming that a participant's utility changed linearly between each of the time points. For participants who died during the trial, their utility was assumed to change linearly between the preceding time point and the time of death, and a value of zero was given to participants from time of death onwards.

### Statistical methods

Our base-case analysis included all participants randomised into the trial except those randomised in error and those who withdrew consent for their data to be used, which is consistent with the main effectiveness analyses. Analyses were performed on an intention-to-treat basis. Overall, 2.5% of resource use data were completely missing, and 10.7% of EQ-5D scores were missing across the three time points in the trial. Missing resource use and EQ-5D data were imputed by multiple imputation using a series of chained regression equations.^18^ Five values were predicted for each missing data cell, and a method called Rubin's Rule was used to summarise data across the five data sets.^19^ Where resource use data were partially missing, for example, for linked questions where only the first part was answered, mean imputation was used. For example, if a participant reported general practitioner visits, but did not record the number of visits, the mean number of visits from other participants was assigned to participants whose data were missing.

Given that baseline utility directly contributes to QALY calculations, it is important to control for any potential imbalances in baseline utility in the estimation of the mean difference in QALYs between treatment groups, to avoid introducing bias.^20^ QALYs were therefore adjusted for baseline EQ-5D. Costs and effects were not discounted as the time horizon was <12 months. The incremental cost-effectiveness ratio (ICER) was derived from the average costs and QALYs gained in each trial group, producing an incremental cost per QALY gained by implementing a restrictive threshold in place of a liberal threshold. Non-parametric bootstrapping of costs and QALYs was then used to quantify the degree of uncertainty around the ICER. A 1000 bootstrap samples were drawn for each of the five imputed data sets.^21^

### Presentation of results

The mean costs and QALYs gained in each trial arm, with SEs and 95% CIs are presented, as well as the ICER. Uncertainty around the ICER is represented graphically on the cost-effectiveness plane by the bootstrap replicates of the mean difference in costs and QALYs between the groups. The restrictive threshold would be considered cost-effective if the ICER falls below £20 000 per QALY, the level below which the National Institute for Health and Care Excellence generally recommends interventions to the NHS; however, the ICERs presented allow decision-makers to assess cost-effectiveness at a willingness-to-pay threshold of their choice.

### Sensitivity analyses

Deterministic sensitivity analyses were used to investigate the impact on the results of the cost and cost-effectiveness analyses when varying key parameters one at a time, or major cost drivers, such as treating costly complications, and also to investigate the impact of uncertainty on the cost-effectiveness results. In terms of costs, key unit costs were varied, the costing was undertaken from the point of randomisation rather than the point of surgery, and the impact of high-cost participants (outliers) were investigated. In terms of outcomes, assumptions for calculating QALYs were varied, and life-years gained was considered as an alternative outcome measure to QALYs.

### Subgroup analyses

Clinical opinion suggests that transfusion decisions should be influenced by patients' characteristics, and that ‘at-risk’ patients should be transfused at a different threshold. Subgroup analyses were conducted to investigate whether cost-effectiveness results varied between the prespecified participant subgroups used for the effectiveness analyses:

- Operation type (isolated coronary artery bypass grafting (CABG) vs other operation types);
- Age at operation (<75 vs ≥75 years);
- Preoperative diagnosis of diabetes (none vs diet, oral medication or insulin controlled);
- Preoperative diagnosis of lung disease (none vs chronic pulmonary disease or asthma);
- Preoperative renal impairment (estimated glomerular filtration rate ≤60 mL/min vs estimated glomerular filtration rate >60 mL/min);
- Sex (males vs females);
- Preoperative ventricular function (good vs moderate or poor).

The impact of subgroups was evaluated using ordinary least squares regression separately for total costs and for QALYs, conditional on treatment group, subgroup and an interaction between treatment group and subgroup, (and baseline EQ-5D for QALYs only).

## Results

The trial randomised a total of 2007 participants; four withdrew, leaving an analysis population consisting of 2003 participants, 1000 in the restrictive group and 1003 in the liberal group. Participants had a mean age of 69 years, and 69% were men. Most participants underwent coronary artery bypass grafting (40.7%) or valve surgery (30.5%).^10^

### Resource use and costs

There was little difference in resource use between the groups (table 1). Red blood cells were the only resource item for which there was a clear difference, an expected finding given that the liberal group by definition had more red blood cells transfused (mean difference 1.00 (SE 0.14) unit per participant). A detailed list of all the unit cost values attached to these resources is provided in the online supplementary appendix 2.

**Table 1:** Resource use per participant to 3 months from surgery

Resource use component	Randomised to restrictive threshold (N=1000) Frequency (%) or mean (SE)	Randomised to liberal threshold (N=1003) **F**requency (%) or mean (SE)	Restrictive vs liberal threshold % or mean (SE) difference
**Red blood cells—**number of units/participant	2.08 (0.09)	3.07 (0.11)	−1.00 (0.14)
**Type of cardiac procedure—**number (%) of participants			
Coronary artery bypass grafting	408 (41)	408 (41)	0
Valve	307 (31)	304 (30)	1
Coronary artery bypass grafting and valve	195 (20)	203 (20)	0
Other	90 (9)	88 (9)	0
**Blood products—**number of units/participant			
Fresh frozen plasma	1.00 (0.06)	0.95 (0.06)	0.05 (0.08)
Platelets	0.65 (0.03)	0.64 (0.03)	0.01 (0.05)
Cryoprecipitate	0.23 (0.03)	0.21 (0.02)	0.02 (0.04)
**Inpatient complications**			
**Primary outcome—**number (%) of participants			
Antibiotics for infectious complication	341 (34)	344 (34)	0
Stroke	14 (1)	16 (2)	−1
Suspected myocardial infarction	3 (0)	7 (1)	−1
Gut infarction	5 (1)	1 (0)	1
Acute kidney injury—stage 3	60 (6)	51 (5)	1
**Other complications—**number of events/participant			
Reoperation	0.09 (0.01)	0.10 (0.01)	−0.01 (0.02)
Reintubation	0.07 (0.01)	0.07 (0.01)	0.00 (0.01)
Tracheostomy	0.03 (0.01)	0.03 (0.01)	0.00 (0.01)
Mask continuous positive airway pressure	0.13 (0.01)	0.12 (0.01)	0.01 (0.02)
Pneumothorax requiring chest drainage	0.01 (0.00)	0.01 (0.00)	0.00 (0.01)
Pleural effusion requiring drainage	0.06 (0.01)	0.06 (0.01)	0.00 (0.01)
Pacing	0.31 (0.02)	0.31 (0.02)	0.00 (0.02)
SVT/AF requiring treatment	0.41 (0.02)	0.39 (0.02)	0.02 (0.03)
VF/VT requiring intervention	0.02 (0.01)	0.01 (0.00)	0.01 (0.01)
Low cardiac output	0.11 (0.01)	0.11 (0.01)	0.00 (0.01)
**Inpatient length of stay—**days/participant			
Cardiac intensive care unit (CICU)	1.14 (0.12)	1.12 (0.13)	0.02 (0.18)
High dependency unit (HDU)	3.09 (0.12)	3.05 (0.12)	0.04 (0.17)
Ward	5.67 (0.15)	5.83 (0.17)	−0.17 (0.23)
Another unit / hospital	1.27 (0.20)	1.36 (0.19)	−0.09 (0.27)
**Blood saving techniques—**number (%) of participants			
Tranexamic acid	807 (81)	810 (81)	0
Trasylol	41 (4)	35 (3)	1
Intraoperative cell salvage	482 (48)	503 (50)	−2
Postoperative cell salvage	56 (6)	46 (5)	1
**Fluids in theatre/CICU/HDU—**number (%) of participants			
Inotropes	624 (62)	614 (61)	1
Gelofusine	843 (84)	836 (83)	1
Hydroxyethyl starch (HES)	231 (23)	233 (23)	0
**Readmissions to hospital**			
Length of stay—days/participant	1.38 (0.15)	1.46 (0.16)	−0.08 (0.22)
**Accident and Emergency (A&E) attendances**			
Total A&E visits—number/participant	0.09 (0.01)	0.08 (0.01)	0.01 (0.01)
**Outpatient appointments—**number/participant			
Cardiac surgery outpatient visits	0.44 (0.02)	0.51 (0.02)	−0.07 (0.03)
Cardiology outpatient visits	0.28 (0.02)	0.26 (0.02)	0.03 (0.03)
Other outpatient visits	0.17 (0.02)	0.17 (0.02)	0.00 (0.03)
**Other healthcare contacts—**number/participant			
General practitioner at surgery	1.99 (0.06)	2.07 (0.07)	−0.09 (0.10)
General practitioner at home	0.43 (0.04)	0.37 (0.03)	0.06 (0.06)
Practice nurse	1.56 (0.13)	1.57 (0.14)	−0.01 (0.19)
District nurse	2.47 (0.20)	2.21 (0.23)	0.26 (0.30)

SVT/AF, supraventricular tachycardia/atrial fibrillation; VF/VT, ventricular fibrillation/ventricular tachycardia.

A breakdown of total costs of care from surgery to 3 months is shown in table 2. Key drivers of total costs were surgery, complications and length of stay (LOS). The difference in the mean units of red blood cells transfused translated into a statistically significant average difference in red blood cell costs (£140, SE 19, p<0.0001). The differences in other cost components between the groups were small, although there was substantial uncertainty around these differences (as is evident from the large SEs). Total costs were £17 945 (SE 332) in the restrictive group and £18 127 (SE 357) in the liberal group, resulting in a mean difference between the groups of −£182 (SE 488; table 2). This difference in cost was largely associated with the higher cost of red blood cells in the liberal group.

**Table 2:** Breakdown of total average cost per participant for both trial groups

Cost component	Randomised to restrictive threshold (N=1000) Mean cost (£) (SE)	Randomised to liberal threshold (N=1003) Mean cost (£) (SE)	Restrictive vs liberal threshold Mean cost (£) difference (SE)
**Red blood cells**	**287 (13)**	**427 (15)**	**−140 (19)**
**Hospital inpatient episode**			
Initial cardiac surgery	7309 (18)	7313 (18)	−4 (26)
Other blood products	206 (12)	199 (11)	7 (16)
Complications and serious adverse events	2684 (137)	2714 (146)	−30 (200)
Length of hospital stay*	5854 (201)	5892 (221)	−38 (299)
Blood saving techniques	159 (9)	152 (8)	7 (12)
Regular medications	26 (2)	29 (2)	−3 (3)
Fluids	55 (1)	55 (1)	0 (2)
**Total**	**16 293 (309)**	**16 353 (339)**	**−60 (459)**
**Postdischarge**			
Hospital readmissions	770 (85)	753 (78)	17 (116)
Accident and Emergency visits	16 (2)	12 (2)	4 (3)
Outpatient appointments	202 (6)	216 (7)	−14 (9)
Other medical/social care	378 (14)	366 (16)	12 (21)
**Total**	**1365 (90)**	**1347 (82)**	**18 (122)**
**Total costs**	**17 945 (332)**	**18 127 (357)**	**−182 (488)**

*Includes days in another unit/hospital once transferred out of the cardiac unit.

### HRQoL and QALYs

There was very little difference in EQ-5D scores between the trial groups at any of the three time points (table 3). On average, participants' EQ-5D scores did not quite return to their preoperative level by 3 months in either treatment group. QALYs to 3 months were 0.180 for both the restrictive and liberal groups, with a mean difference of only 0.0004 (SE 0.0021; table 3). This difference of 0.0004 QALYs is ∼3.5 quality-adjusted hours. Although there was a significant difference in deaths between the groups in favour of the liberal group, this did not translate into a difference in QALYs between the groups. Exploratory plots of the QALY data for survivors and non-survivors at 3 months revealed that it was not just participants who died who had low QALYs, but also many other participants, hence the difference in deaths did not have a major impact on the quality-of-life results.

**Table 3:** Results for EQ-5D scores and QALYs

	Randomised to restrictive threshold (N=1000) Mean (SE)	Randomised to liberal threshold (N=1003) Mean (SE)	Restrictive vs liberal threshold Mean difference (SE)
EQ-5D time point*			
Baseline	0.765 (0.008)	0.767 (0.007)	−0.001 (0.011)
6 weeks	0.692 (0.008)	0.686 (0.008)	0.006 (0.011)
3 months	0.748 (0.009)	0.750 (0.008)	−0.002 (0.012)
**QALYs to 3 months** (adjusted for baseline EQ-5D)	0.1802 (0.0015)	0.1798 (0.0016)	0.0004 (0.0021)

*Deaths included as zero.

QALY, quality-adjusted life year.

### Cost-effectiveness

**Figure 1:**
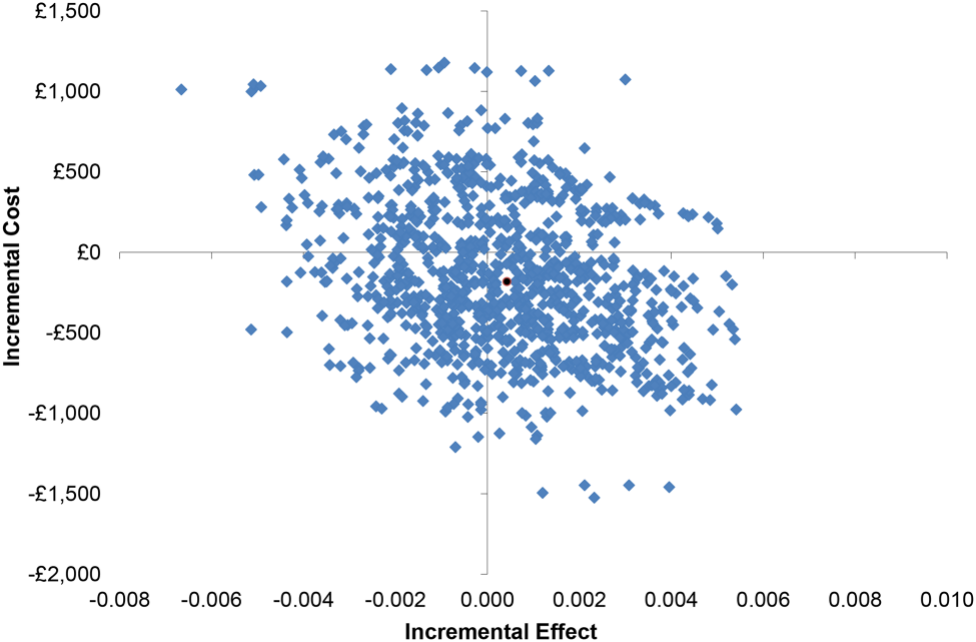
Cost-effectiveness plane. In order that the points could be seen, only 1000 replicates were plotted (200 replicates for each of the five imputations).

When we considered the point estimate (the initial mean estimate), the restrictive threshold is considered cost-effective: the restrictive threshold is dominant over the liberal threshold, since it is both more effective and less costly (table 4). However, there is great uncertainty around this result, as shown on the cost-effectiveness plane in figure 1. The differences in costs and QALYs between the groups are incredibly small, and therefore the point estimate (the black dot) is close to the origin. The bootstrap replicates of the cost and QALY differences cover all four quadrants of the cost-effectiveness plane, which illustrates that there is actually very little difference between the two groups along with much uncertainty. There is a 43% probability that the restrictive threshold dominates the liberal threshold, but also a 20% probability of the reverse scenario, that the liberal threshold dominates the restrictive threshold. In reality, there is no difference in QALYs between the groups, the 95% CI suggests the maximum difference is less than ± 2 days.

**Table 4:** Base case cost-effectiveness results

Total costs (95% CI)			QALYs (95% CI)			ICER	Probability restrictive is cost-effective at a ceiling ratio of		
Restrictive threshold (N=1000)	Liberal threshold (N=1003)	Restrictive vs liberal threshold	Restrictive threshold (N=1000)	Liberal threshold (N=1003)	Restrictive vs liberal threshold	Cost/QALY	£20 000	£50 000	£100 000
£17 945 (£17 273 to £18 618)	£18 127 (£17 450 to £18 804)	−£182 (−£1108 to £744)	0.1802 (0.1772 to 0.1832)	0.1798 (0.1766 to 0.1829)	0.0004 (−0.0037 to 0.0045)	Restrictive dominant (−£428 064)	65%	66%	66%

ICER, incremental cost-effectiveness ratio; QALY, quality-adjusted life year.

95% CIs are based on parametric methods, using SEs from the bootstrap replicates and a t distribution with degrees of freedom v=(M−1) (1+r^−1^)^2^, where r is the ratio of the between-imputation component of the variance and the within-imputation component of the variance, and M is the number of imputed data sets.^22^

### Sensitivity analyses

Sensitivity analyses (see online supplementary appendix 3) demonstrated that the conclusions were sensitive to a few very high-cost participants, but robust to changes in unit costs, to moving the time origin from surgery to the time of randomisation, to alternative assumptions for calculating QALYs, and to using life-years instead of QALYs as an alternative outcome measure.

The distribution of total costs per participant was positively skewed for both groups. This skewness was enhanced by the existence of a few very high-cost outliers, especially in the liberal group. Four participants, all in the liberal group, had costs over £100 000 (£101 173; £107 163; £108 865 and £144 985, compared to the average costs of £18 127). These participants did not have unexpected events; rather, they had large numbers of expected complications and stayed in hospital with a high level of care for some time. Therefore, there were no grounds for excluding these participants from our analyses. Nevertheless, it is instructive to investigate the impact they are having on results since the imbalance across groups of these outliers could easily have arisen by chance. If the participant with the highest cost is excluded, the difference in costs between the groups reduces from −£182 to −£55 (smaller than the cost of the average difference in red blood cell use). If participants with the four highest costs are excluded, the liberal group becomes less expensive than the restrictive group, and the difference in costs between the groups changes from −£182 to +£208. It is clear that these four participants exerted a significant impact on the average costs of participants in the liberal group.

### Subgroup analyses

There was evidence of only one subgroup effect: participants in the restrictive group with chronic pulmonary disease or asthma had slightly less QALYs compared to other participants (p=0.003). See online supplementary appendix 4 for more details.

## Discussion

There was very little difference between the alternative transfusion groups in either costs or effects, and great uncertainty around the cost-effectiveness results. When a breakdown of total costs was considered, there was a clear difference in the costs associated with red blood cells between the two groups as expected, because the liberal group was given more red blood cells by definition; otherwise, cost components were very similar. The differences in costs between groups were about the same when considering only the red blood cell costs and when considering all costs; however, the former difference was estimated more precisely than the latter. Mean QALYs to 3 months were effectively identical in both groups. The point estimate of cost-effectiveness suggested that the restrictive group was more effective (very slightly greater QALY gain) and less costly than the liberal group (ie, dominant), and therefore cost-effective. However, the extreme uncertainty around this result makes the point estimate less informative. The uncertainty is shown on the cost-effectiveness plane by the position of the point estimate close to the origin, and by the fact that the bootstrap replicates of the cost and QALY differences covered all four quadrants of the plane. Moreover, there were several outliers in the liberal group which exerted a substantial influence on the average costs of participants in that treatment group, reversing the direction of the results described above when they were excluded.

Our economic evaluation had several key strengths. It was based on high-quality cost and quality of life individual patient data collected in a randomised trial. Very detailed data collection was undertaken, and the trial achieved excellent completeness of follow-up (follow-up at 3 months postrandomisation was obtained for 98.7% of participants). TITRe2 was a large trial, randomising four times more participants than the next largest trial comparing restrictive and liberal transfusion thresholds after cardiac surgery. In TITRe2, only participants who breached the liberal threshold were randomised; this avoided diluting the treatment effect with similar numbers of participants in each group who were unlikely to be transfused. Costs and cost-effectiveness estimates were similarly not diluted by patients who were unlikely to be transfused. There is one limitation to note around the unit costs of administering blood products. The unit costs of administering red blood cells used in this study captured the nursing costs associated with transfusion (based on UK data), but not other inputs such as those of the transfusion laboratory; and no costs associated with administration were included for other blood products. Unit costs for blood administration used here were much lower than those reported elsewhere.^23–25^ Given there was only a difference of one unit of red blood cells between the transfusion groups, (and a very small difference in total costs), it is unlikely that the inclusion of additional blood administration costs would alter the conclusions.

To the best of our knowledge, this is the first trial-based economic evaluation assessing the cost-effectiveness of alternative transfusion thresholds after cardiac surgery. A Cochrane systematic review of randomised controlled trials (RCTs) comparing restrictive and liberal transfusion thresholds in surgical patients and the critically ill was published in 2012.^26^ None of the RCTs included in the review, nor five additional trials published subsequently, included an integral economic evaluation,^27–31^ (one pilot trial included an exploratory economic evaluation, but the only inpatient resource captured was LOS by level of care).^31^

Although the TITRe2 trial was a well-designed and rigorously conducted trial, the interpretation of its cost-effectiveness results is challenging because the differences in costs and quality of life between the groups were small or uncertain (across both the clinical and cost-effectiveness analyses). Point estimates of cost-effectiveness (based on QALYs as the primary health economic outcome measure or life years in a sensitivity analysis) suggested that a restrictive threshold was cost-effective, but there was extreme uncertainty around these results. From an economic perspective, we conclude that there is no difference between the restrictive and liberal groups. While there was no difference in the primary clinical outcome, there was a borderline difference in mortality between the groups, favouring a liberal threshold. It is difficult to recommend restrictive transfusion after cardiac surgery, given that more patients died in the restrictive group. While there is a growing body of evidence that restrictive transfusion thresholds are safe for most patient groups,^32^ there is recognition that patients with acute or chronic cardiovascular disease, may benefit from more liberal transfusion.^33^ ^34^

A difference of ∼£200 between the groups is a modest cost difference (∼1% of total costs). However, since 34 174 cardiac surgery procedures were undertaken in the UK in 2012/2013,^35^ a difference of £200 in each procedure would have resulted in savings or additional costs of £6.8 million for the NHS. The effect of this cost difference, and whether it is a cost saving or additional cost, is clearly important for the NHS. If there is a saving of ∼£200 per patient, and this is largely attributable to savings in the cost of blood, this would substantially reduce the amount paid to NHSBT for blood products. These savings could be used to support Patient Blood Management (PBM) initiatives, to optimise care for patients who might need a transfusion. A recent audit of PBM in surgery highlighted considerable variation in practice, and the need for hospitals to develop single-unit transfusion policies with clearly defined transfusion triggers.^36^

In summary, our findings suggest that there is no health economic evidence to suggest a difference between the two alternative blood transfusion thresholds, as there was very little difference between the alternative transfusion groups in either costs or effects, but great uncertainty around the cost-effectiveness results.
