# Supplementary material for: Are lower levels of red blood cell transfusion more cost-effective than liberal levels after cardiac surgery? Findings from the TITRe2 randomised controlled trial
Source: BMJ Open. 2016 Aug 1;6(8):e011311. doi: 10.1136/bmjopen-2016-011311 (PMC4985876; doi:10.1136/bmjopen-2016-011311)
Supplement: Supplementary appendices [file bmjopen-2016-011311supp_appendices.pdf]

<u>Appendix 1. Stokes et al. TITRe2 Cost-effectiveness paper</u>

CONSORT diagram summarising TITRe2 trial design.

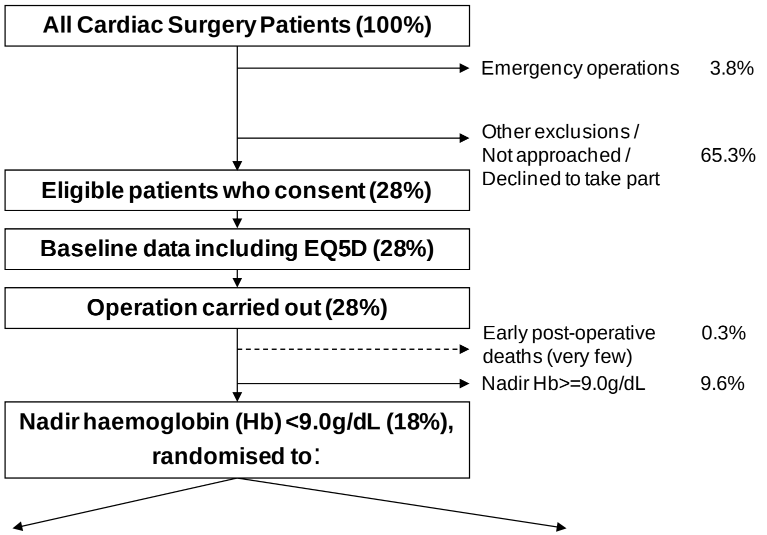

Transfusion threshold: Hb <9.0g/dL

	TRANSFUSION THRESHOLD		
	Nadir Hb <7.5g/dL	Nadir Hb ≥7.5 to <9.0 g/dL	Total
TRANSFUSED	96%	62%	74%
NOT TRANSFUSED	4%	38%	26%

Transfusion threshold: Hb <7.5g/dL

	TRANSFUSION THRESHOLD		
	Nadir Hb <7.5g/dL	Nadir Hb ≥7.5 to <9.0 g/dL	Total
TRANSFUSED	96%	<5%	<35%
NOT TRANSFUSED	4%	>=95%	>=65%

Notes:

Percentages are based on data from the cardiac surgery registry in Bristol for the period January to September 2007. An unknown percentage of patients are excluded by the exclusion criteria because the registry does not contain sufficient detail to apply the definitions proposed for the trial. However, patients meeting one or more of these criteria are extremely rare and we expect all of the exclusion criteria to account for a maximum of 5% of cardiac surgery patients.

Hb, haemoglobin.

Appendix 2. Stokes et al. TITRe2 Cost-effectiveness paper

Table 1 Unit costs for surgery, inpatient stays, and blood products

Resource	Unit cost (£)	Reference
**Cardiac surgery and reoperations**		
CABG	6714	NHS Reference Costs 2012/13.[1] Elective inpatients. HRG code EA14 for service codes 170 (Cardiothoracic surgery) and 172 (Cardiac surgery). For each code, the costs associated with the average LOS reported were subtracted at a cost of £392 per day (see 'Cardiac ward stay'), and £227 was subtracted for blood products based on data from an audit of blood transfusion in cardiac surgery.[2] An average cost for the codes was then generated, weighted by activity.
Valve	7336	NHS Reference Costs 2012/13.[1] Elective inpatients. HRG codes EA17 (single valve) and EA52 (more than 1 valve) for service codes 170 and 172. For each code, the costs associated with the average LOS reported were subtracted at a cost of £392 per day, and £659 was subtracted for blood products.[2] An average cost for the codes was then generated for single and more than 1 valve procedures, weighted by activity. Finally these 2 figures were weighted to produce an average that reflects the proportion of single valve procedures in TITRe2 participants (90% single, 10% more than 1 valve).
CABG and valve	8054	NHS Reference Costs 2012/13.[1] Elective inpatients. HRG code EA51 for service codes 170 and 172. For each code, the costs associated with the average LOS reported were subtracted at a cost of £392 per day, and £1421 was subtracted for blood products.[2] An average cost for the codes was then generated, weighted by activity.
Other	8298	NHS Reference Costs 2012/13.[1] Elective inpatients. HRG code EA20 for service codes 170 and 172. For each code, the costs associated with the average LOS reported were subtracted at a cost of £392 per day, and £1421 was subtracted for blood products.[2] An average cost for the codes was then generated, weighted by activity.
Reoperations <3 hours, excluding blood and LOS	6608	As 'Other' cardiac procedure above, but the lower quartile unit cost was used rather than the mean cost.
Reoperations <3 hours, including blood, excluding LOS	8029	As 'Reoperations <3 hours, excluding blood and LOS', with £1421 for blood products added back in.
Reoperations ≥3 hours, excluding	8298	As 'Other' cardiac procedure above.

Resource	Unit cost (£)	Reference
blood and LOS		
Reoperations ≥3 hours, including blood, excluding LOS	9719	As ‘Other’ cardiac procedure above, with £1421 for blood products added back in.
**Inpatient stay**		
Cardiac ward day	392	NHS Reference Costs 2012/13.[1] Weighted average of elective inpatient excess bed day costs for relevant HRGs (EA14, EA16, EA17, EA19, EA20, EA22, EA51, EA52, excluding any service codes for paediatrics).
HDU day	619	NHS Reference Costs 2012/13.[1] Critical Care Services - Adult: Critical Care Unit (XC07Z, 0 organs supported).
CICU day	1190	NHS Reference Costs 2012/13.[1] Critical Care Services - Adult: Critical Care Unit (weighted average of XC01Z - XC06Z, 1-6 organs supported).
General ICU day	1608	NHS Reference Costs 2012/13.[1] Critical Care Services - Adult: Critical Care Unit (weighted average of XC01Z - XC03Z, 4-6 organs supported).
Ward day for another unit in the hospital, or at another hospital	265	NHS Reference Costs 2012/13.[1] Non-elective inpatient excess bed day cost across all activities.
**Blood products**		
Red blood cells	123.31	NHSBT Price List 2012/13[3]
RBC administration cost, first unit	22	Primary data collection of the nursing time and consumables associated with requesting blood and administering transfusions undertaken with collaborators on the 'Trial of prophylactic versus no prophylactic platelet transfusions' (TOPPS) trial, funded by NHSBT. Preliminary analyses show it takes 49 minutes of nursing time and £6 of consumables to request and administer the first unit of RBCs.
RBC administration cost, subsequent units	5	As above; analyses found it took 15 minutes of nursing time to administer subsequent units (no additional consumables).
Fresh frozen plasma	27.46	NHSBT Price List 2012/13[3]
Platelets	209.30	NHSBT Price List 2012/13[3]
Cryoprecipitate	189.19	NHSBT Price List 2012/13[3]

CABG, Coronary artery bypass grafting; CICU, Cardiac intensive care unit; HDU, High dependency unit; HRG, Healthcare resource group; ICU, Intensive care unit; LOS, Length of stay; NHSBT, NHS Blood and Transplant; RBC, Red blood cell

Table 2 Unit costs for blood saving techniques, fluids and medications in theatre/CICU/HDU

Resource	Assumed quantity	Unit cost^{+} (£)	Reference
Tranexamic acid	5g intravenously	15.50	BNF 66[4]
Aprotinin	6 million KIU intravenously	316.83	Davies, Brown, Haynes et al.[5] using data from BNF 47.[6] Costs have been inflated using the HCHS inflation index.
Intra- / post-operative cell salvage		176	Davies, Brown, Haynes et al.[5] Costs have been inflated using the HCHS inflation index.
Activated Factor VII	5mg intravenously	2486.60	Oxford University Hospitals NHS Foundation Trust, 2013
Beriplex® (CSL Behring UK Ltd)	1500 IU intravenously	420	District General Hospital, South Central, 2014
Hydroxyethyl starch (HES)	1500ml intravenously	40.60	BNF 58.[7] Costs have been inflated using the HCHS inflation index.
Human albumin solution	500ml intravenously	36	District General Hospital, South Central, 2014
Gelatin (Gelofusine, B. Braun Medical Ltd)	1500ml intravenously	7.92	Teaching Hospital, South Central, 2013
Inotropes	Noradrenaline 1mg/hour for 5 days	57.30	eMIT, 2014[8]
Hartmann's solution	1500ml intravenously	2.67	Teaching Hospital, South Central, 2013
Gelatin (Isoplex, Beacon Pharmaceuticals)	1500ml intravenously	22.07	BNF 66[4]

BNF, British National Formulary; CICU, Cardiac intensive care unit; eMIT, electronic Marketing Information Tool; HCHS, Hospital and community health services; HDU, High dependency unit; KIU, Kallikrein Inhibitor Units

Table 3 Unit costs for antibiotics

Drug name	Route	Assumed dose/frequency per day	Daily cost (£)	Source	Daily cost (£) from BNF[4] for sensitivity analyses
Amikacin	IV	500mg 3xday	18.42	eMIT[8]	
Amoxicillin	Oral	500mg 4xday	0.10	eMIT[8]	0.31
Amoxicillin	IV	500mg 4xday	1.46	eMIT[8]	2.20
Aztreonam	IV	1g 3xday	28.20	BNF[4]	
Benzyl-penicillin	IV	1.2g 4xday	7.56	BNF[4]	
Caspofungin	IV	70mg first day, 50mg 1xday subsequent days	416.78 day 1, 327.67 thereafter	BNF[4]	
Cefalexin	Oral	250mg 4xday	0.11	eMIT[8]	
Cefotaxime	IV	1g 2xday	1.22	eMIT[8]	
Ceftazidime	IV	1g 3xday	2.56	eMIT[8]	
Ceftriaxone	IV	1g 1xday	0.53	eMIT[8]	
Cefuroxime	IV	750mg 3xday	1.37	eMIT[8]	
Cefuroxime 1.5g	IV	1.5g 3xday	2.13	eMIT[8]	
Cefuroxime 750mg	IV	750mg 3xday	1.37	eMIT[8]	
Chloramphenicol	IV	1g 4xday	5.56	BNF[4]	
Ciprofloxacin	Oral	500mg 2xday	0.05	eMIT[8]	0.15
Ciprofloxacin	IV	400mg 2xday	2.02	eMIT[8]	39.58
Clarithromycin	Oral	250mg 2xday	0.13	eMIT[8]	
Clarithromycin	IV	500mg 2xday	5.24	eMIT[8]	
Clindamycin	Oral	150mg 4xday	0.24	eMIT[8]	
Clindamycin	IV	600mg 3xday	7.27	eMIT[8]	

Drug name	Route	Assumed dose/frequency per day	Daily cost (£)	Source	Daily cost (£) from BNF[4] for sensitivity analyses
Co-amoxiclav	Oral	375mg 3xday	0.21	eMIT[8]	0.32
Co-amoxiclav 625mg	Oral	625mg 3xday	0.22	eMIT[8]	
Co-amoxiclav	IV	600mg 3xday	1.64	eMIT[8]	3.63
Co-amoxiclav 1.2g	IV	1.2g 3xday	1.91	eMIT[8]	
Colistimethate sodium	Oral	1 million units 2xday	3.36	BNF[4]	
Colistimethate sodium	IV	1 million units 2xday	3.36	BNF[4]	
Co-trimoxazole	Oral	960mg 2xday	0.49	eMIT[8]	
Co-trimoxazole	IV	960mg 2xday	7.12	BNF[4]	
Daptomycin	IV	350mg 1xday	62.00	BNF[4]	
Demeclocycline	Oral	150mg 4xday	11.64	BNF[4]	
Doxycycline	Oral	200mg first day, 100mg 1xday subsequent days	0.07 day 1, 0.03 thereafter	eMIT[8]	0.28 day 1, 0.14 thereafter
Ertapenem	IV	1g 1xday	31.65	BNF[4]	
Erythromycin	Oral	250mg 4xday	0.11	eMIT[8]	0.24
Erythromycin	IV	Erythromycin lactobionate 1g 4xday	43.92	BNF[4]	
Flucloxacillin	Oral	250mg 4xday	0.11	eMIT[8]	
Flucloxacillin	IV	0.25g 4xday	1.68	eMIT[8]	4.92
Fluconazole	Oral	400mg first day, 200mg 1xday subsequently	0.19 day 1, 0.09 thereafter	eMIT[8]	
Fluconazole	IV	400mg first day, 200mg 1xday subsequently	1.80 day 1, 0.94 thereafter	eMIT[8]	
Fusidic acid	Oral	500mg 3xday	1.81	BNF[4]	
Gentamicin	IV	80mg 3xday	1.57	eMIT[8]	5.85

Drug name	Route	Assumed dose/frequency per day	Daily cost (£)	Source	Daily cost (£) from BNF[4] for sensitivity analyses
Imipenem	IV	500mg 4xday	17.67	eMIT[8]	
Levofloxacin	Oral	500mg 1xday	0.23	eMIT[8]	
Levofloxacin	IV	500mg 1xday	1.87	eMIT[8]	
Linezolid	Oral	600mg 2xday	89.00	BNF[4]	
Linezolid	IV	600mg 2xday	89.00	BNF[4]	
Meropenem	IV	0.5g 3xday	7.86	eMIT[8]	24.00
Metronidazole	Oral	400mg 3xday	0.05	eMIT[8]	0.21
Metronidazole	IV	500mg 3xday	1.20	eMIT[8]	9.30
Nitrofurantoin	Oral	50mg 4xday	5.23	BNF[4]	
Oseltamivir	Oral	75mg 2xday	3.08	BNF[4]	
Piperacillin/tazobactam	IV	4.5g 3xday	5.68	eMIT[8]	
Rifampicin	Oral	300mg 3xday	0.42	eMIT[8]	
Rifampicin	IV	600mg 3xday	7.66	eMIT[8]	
Teicoplanin	IV	400mg 2xday for 3 doses, subsequently 400mg 1xday	12.24 day 1, 6.12 thereafter	eMIT[8]	14.64 day 1, 7.32 thereafter
Temocillin	IV	1g 2xday	50.90	BNF[4]	
Timentin	IV	3.2g 3xday	15.99	BNF[4]	
Trimethoprim	Oral	200mg 2xday	0.03	eMIT[8]	0.14
Vancomycin	IV	0.5g 2xday	2.32	eMIT[8]	12.50

BNF, British National Formulary; eMIT, electronic Marketing Information Tool; IV, Intravenous

Table 4 Unit costs for regular medications

Recorded on CRF	Assumed drug	Assumed route	Assumed dose/frequency per day	Daily cost (£) from BNF[4]
Digoxin	Digoxin	Oral	125mcg daily	0.04
Diuretics	Furosemide	Oral	40mg daily	0.03
Beta blockers	Atenolol	Oral	25mg daily	0.03
Calcium antagonists	Amlodipine	Oral	5mg daily	0.03
Aspirin	Aspirin	Oral	75mg daily	0.03
Oral nitrates	Isosorbide dinitrate	Oral	80mg daily	0.98
Angiotensin 2 blockers	Losartan	Oral	25mg daily	0.04
ACE inhibitors	Ramipril	Oral	5mg daily	0.04
Warfarin	Warfarin sodium	Oral	3mg daily	0.03
Clopidogrel	Clopidogrel	Oral	75mg daily	0.06
Statins	Simvastatin	Oral	40mg daily	0.04
Anti-arrhythmic	Amiodarone	Oral	200mg daily	0.06
Heparin/clexane	Enoxaparin sodium	S/C	20mg daily	2.27
IV GTN/nitrates	Glyceryl trinitrate	IV	25mg daily	6.49
$FeSO_4$	Ferrous sulphate	Oral	200mg (65mg iron) 3xday	0.11

ACE, Angiotensin converting enzyme; BNF, British National Formulary; CRF, Case report form; $FeSO_4$, Ferrous sulphate; GTN, Glyceryl trinitrate; IV, Intravenous; S/C, Subcutaneous

Table 5 Resource use assumed for complications and total costs

Complication	Treatment/Action	Cost (£)	Assumptions
Sepsis	No additional treatment	0	Antibiotics recorded separately and costed
Wound infection	No additional treatment	0	Antibiotics recorded separately and costed

Complication	Treatment/Action	Cost (£)	Assumptions
Permanent stroke	Rehabilitation (plus scan)	139	
	CT scan	62	
	MRI scan	248	
Suspected MI	Emergency angiography, transthoracic echo, ECG	1868	
Gut infarction	CT scan	62	
	If confirmed by laparotomy	2693	
AKI - stage 3 only	Haemofiltration	1438	Assume treatment for 2 days
TIA	CT scan	62	
Pancreatitis	CT scan, parenteral nutrition, IV fluids	275.49	Reoperations already captured
Intestinal obstruction/perforation	Laparotomy, parental nutrition	2893	Reoperations already captured
Post-operative haemorrhage	Chest x-ray	41	Reoperations already captured. Assume no additional costs for participants who have a re-operation on the same day/following day as post-operative haemorrhage.
ARDS	Transoesophageal echo, 3 chest x-rays	395	Re-intubation and intensive care already captured
Re-intubation/ventilation	Transoesophageal echo, 3 chest x-rays	395	
Initiation of mask CPAP	CPAP, chest x-ray	539	
Tracheostomy	Tracheostomy, chest x-ray	5354	
Pneumothorax requiring chest drainage	Chest x-ray, chest drain	4218	
Pleural effusion requiring drainage	Chest x-ray, chest drain	4218	
Pacing	Temporary pacemaker	3073	
SVT/AF requiring treatment	Amiodarone	4.79	
Deep vein thrombosis	Duplex scan of leg veins, intraveneous heparin	202.43	Warfarin already captured

Complication	Treatment/Action	Cost (£)	Assumptions
VF/VT requiring intervention	Transoesophageal echo, emergency coronary angiography, chest x-ray	2007	Emergency re-operations and re-intubation captured elsewhere
Low cardiac output requiring management (including IABP)	Transoesophageal echo, chest x-ray	313	
Wound dehiscence requiring rewiring/treatment	Minor treatment (£161), or Negative Pressure Wound Therapy (NPWT) therapy if stated (£3501)	161 or 3501	Assume reoperation covers this complication if reoperation the same day or next day
Cardiac tamponade	Transoesophageal echo, chest x-ray	313	Reoperations and RBCs already captured
Other GI complications			
Abominal distention/small bowel dilation/abdominal pain	CT scan	62	
Coffee ground vomitus	Omeprazole	12.68	
Colonic pseudo-obstruction	CT scan	62	
Constipation	Laxatives, enemas	2.25	
Diabetes inference/exacerbation	Insulin	138.60	
Diagnostic laparotomy	Laparotomy	2693	
Diarrhoea/diarrhoea and vomiting	Isolation room, stool culture and IV for dehydration	3592	
Duodenal ulcer	Endoscopy	676	
Dysphagia/poor swallow/difficult chewing with hoarse voice	Speech and language therapy review, nasendoscopy, oropharyngeal fluoroscopy and maybe CT head	370	
Gastric bubble	Nasogastric tube insertion	252	
Gastritis, Gastro oesophageal reflux	Omeprazole	12.68	

Complication	Treatment/Action	Cost (£)	Assumptions
GI bleed	Endoscopy	676	
GI bleed - duodenal ulcer	Endoscopy and in severe cases laparotomy	2022	
Haematemesis	Omeprazole	12.68	
Hepatic impairment	CT scan	62	
Ileus/gallstone ileus/paralytic ileus	CT scan	62	
Intestinal ischaemia	Laparotomy, CT scan	2755	
Ischaemic bowel and GI bleed	Laparotomy, CT scan	2755	
Laproscopy	Laproscopy	2693	
Melaena	Endoscopy, omeprazole	688.68	
Melaena and bleeding duodenal ulcers	Endoscopy, omeprazole	688.68	
Nausea and/or vomiting	Anti-nausea medication	0.63	
Nasogastric tube inserted	Nasogastric tube inserted	252	
Not absorbing due to abdominal aortic aneurysm repair	CT scan	62	
Rectal bleed	Endoscopy	676	
Upper GI bleed, due to transoesophageal echo	Endoscopy	676	
Other pulmonary complications – all assumed to have 2 chest x-rays, add £82 to all			
Suspected chest infection	No additional treatment	0	Antibiotics and re-intubation already captured
Aspiration pneumonia/pneumonia	No additional treatment	0	Antibiotics and re-intubation already captured
Bilevel positive airway pressure (BIPAP) commenced	BIPAP	498	

Complication	Treatment/Action	Cost (£)	Assumptions
Basal atelectasis	Chest x-ray, physiotherapy	180	CPAP already captured
Basal respiratory wheeze	Nubulised 0.9% saline (10ml) or salbutamol (2.5 mg) 4 times daily	1035	
Bronchopneumonia	No additional treatment	0	Antibiotics and re-intubation already captured
Fluid overload	No additional treatment	0	Antibiotics and re-intubation already captured
Left and right haemothorax/haemothorax requiring chest drainage	Chest drain	4177	
Heart failure	Diuretics	0.15	Re-intubation already captured
Increasing oxygen requirements, chest examination	Physiotherapy	139	CPAP and re-intubation already captured
Infection	No additional treatment	0	Antibiotics and re-intubation already captured
Left pneumothorax	Chest x-ray	41	
Lower respiratory tract infection	No additional treatment	0	Antibiotics and re-intubation already captured
Overloaded	Diuretics	0.15	Re-intubation already captured
Pericardial effusion/pericardial effusions with atelectasis	No treatment in mild cases	0	Reoperation and re-intubation already captured
Pleuritic pain	Analgesia	6	
Pneumonia and respiratory failure	No additional treatment	0	Antibiotics and re-intubation already captured
Pulmonary oedema	Diuretics	0.15	Re-intubation already captured
Right lower lobe collapse and atelectasis	Chest x-ray, physiotherapy	180	CPAP and re-intubation already captured

Complication	Treatment/Action	Cost (£)	Assumptions
changes on chest x-ray			
Reduced air entry to bases/respiratory distress/respiratory failure	Physiotherapy	139	CPAP and re-intubation already captured
Respiratory arrest	No additional treatment	0	Re-intubation already captured
Slight decrease in entry in both bases	Physiotherapy	139	CPAP and re-intubation already captured
(Small) pleural effusion left side/right side/bilateral	No treatment	0	
Surgical emphysema	Chest drain, chest x-ray	4218	
Aspirated on nasogastric tube insertion	Nasogastric tube insertion (if not already included)	252	
Disconnected chest drain	Chest drain	4177	
Increased pulmonary artery pressure	No treatment	0	
Left basal effusion and right basal collapse	Physiotherapy	139	CPAP and re-intubation already captured
Other arrhythmia complications – all assumed to have 2 ECGs, add £106 to all			
AV block/1st degree heart block/third degree AV block/complete heart block	No additional treatment	0	Pacing already captured
First degree heart block with aterial ectopics	No treatment	0	
Complete heart block, permanent pacemaker	Permanent pacemaker	14564	
Heart block-paced	No additional treatment	0	Pacing already captured
Arrhythmia	Amiodarone	4.79	
Asystole/P wave asystole/cardiac arrest/Pulseless electrical activity arrest	CPR	1491	Re-intubation already captured

Complication	Treatment/Action	Cost (£)	Assumptions
Asystole with permanent pacemaker insertion	Permanent pacemaker	14564	
Atrial Flutter	Amiodarone	4.79	
Bradycardia/intermittant bradycardia/nodal bradycardia/sinus bradycardia	No additional treatment	0	Pacing already captured
Bradycardic episode with left bundle branch block, 24 hour tape performed	24 hour holter monitor	204	
Cardioversion	Cardioversion	808	
Heart rate irregular, commenced on amiodarone	Amiodarone	4.79	
Ectopics/multiple ectopics/ventricular ectopics	No treatment	0	
Fast AF requiring amiodarone and pacing switched off	Amiodarone	4.79	
Junctional rhythm	No additional treatment	0	Pacing already captured
Left bundle branch block/new onset of left branch block/right bundle branch block	No treatment	0	
Loss of cardiac output	CPR	1491	Re-intubation already captured
New AF	No additional treatment	0	Pacing already captured
Permanent pacemaker implanted	Permanent pacemaker	14564	
SVT/Flutter	Amiodarone	4.79	
Sinus tachycardia	No treatment	0	
Type B Wolf Parkinson White pattern	No treatment	0	
Sinus pauses	No additional treatment	0	Pacing already captured
Vasovagal episode	No treatment	0	

Complication	Treatment/Action	Cost (£)	Assumptions
Other thromboembolic complications			
CT head confirmed occipital infarction	CT scan	62	
Cerebral infarct	CT scan	62	
Saphenous vein graft thrombosed to right coronary artery	Coronary angiography	1694	
Thrombophlebitis	Analgesia	6	

AKI, Acute kidney injury; ARDS, Acute respiratory distress syndrome; AF, Atrial fibrillation; AV, Atrioventricular; BIPAP, Bilevel positive airway pressure; CPAP, Continuous positive airway pressure; CPR, Cardiopulmonary resuscitation; CT, Computed tomography; ECG, Electrocardiography; GI, Gastrointestinal; IABP, Intra-aortic balloon pump; IV, Intravenous; MI, Myocardial infarction; MRI, Magnetic resonance imaging; NPWT, Negative pressure wound therapy; RBC, Red blood cell; SVT, Supraventricular tachycardia; TIA, Transient ischemic attack; VF/VT, Ventricular fibrillation/ Ventricular tachycardia

Notes:

Resource use assumed for complications and total costs are shown here, unit costs and sources are shown in the next table.

Table 6 Unit costs for complications

Treatment/Action	Unit cost+ (£)	Reference
24 hour holter monitor	204	NHS Reference Costs 2012/13.[1] Day Cases. EA47Z Electrocardiogram Monitoring and stress testing. 320 Cardiology. Lower quartile cost.
Antibiotics (piperacillin/tazobactam, 4.5g IV 3xday for 5 days)	28.40	eMIT[8]
Amiodarone (1.2g IV, then oral 200mg 3xday for 1 week, 2xday for 1 week)	4.79	eMIT[8]
Analgesia (morphine sulphate, 10mg IV every 4 hours for 5 days)	6	eMIT[8]
Anti-nausea medication (ondanestron, 4mg IV for 5 days)	0.63	eMIT[8]
BIPAP	498	As CPAP
Cardioversion	808	Lord, Willis, Eatock et al.[9] Costs have been inflated using the HCHS inflation index.
Chest drain	4177	NHS Reference Costs 2012/13.[1]
Chest x-ray	41	Teaching Hospital, South Central, 2012. Costs have been inflated using the HCHS inflation index.
Coronary angiography	1694	NHS Reference Costs 2012/13.[1]
CPAP	498	Gray, Goodacre, Newby et al.[10] Costs have been inflated using the HCHS inflation index.
CPR	1491	NHS Reference Costs 2012/13.[1]
CT scan	62	NHS Reference Costs 2012/13.[1] Diagnostic Imaging - Direct Access. RA08A Computerised Tomography Scan, one area, no contrast, 19 years and over. 100 General surgery.

**Treatment/Action**	**Unit cost⁺ (£)**	**Reference**
Diuretics (furosemide 40mg orally for 5 days)	0.15	BNF[4]
Drainage of pus under local anaesthesia	426	NHS Reference Costs 2012/13.[1] Elective inpatients. JC43A Minor Skin Procedures, 13 years and over. 320 Cardiology
Drainage of pus under general anaesthesia	1919	NHS Reference Costs 2012/13.[1] Elective inpatients. JC43A Minor Skin Procedures, 13 years and over. 172 Cardiac Surgery
Duplex scan of leg veins	155	NHS Reference Costs 2012/13.[1] Diagnostic Imaging – Outpatients. RA10Z Computerised Tomography Scan, one area, pre and post contrast. 172 Cardiac Surgery
ECG	53	NHS Reference Costs 2012/13.[1] Directly Accessed Diagnostic Services. EA47Z Electrocardiogram Monitoring and stress testing.
Echo - transthoracic	121	NHS Reference Costs 2012/13.[1] Diagnostic Imaging – Outpatients. RA60A Simple Echocardiogram, 19 years and over. 172 Cardiac Surgery
Echo - transoesophageal	272	NHS Reference Costs 2012/13.[1] Day Cases. EA45Z Complex Echocardiogram, including Transoesophageal and Fetal Echocardiography. 320 Cardiology. Lower quartile cost.
Endoscopy	676	NHS Reference Costs 2012/13.[1]
Fluoroscopy	115	NHS Reference Costs 2012/13.[1] Diagnostic Imaging – Outpatients. RA16Z Contrast Fluoroscopy Procedures, less than 20 minutes. 172 Cardiac Surgery
Haemofiltration (assume for 2 days)	1438	NHS Reference Costs 2012/13.[1] Renal Dialysis at Base. LE01A. Haemodialysis for Acute Kidney Injury, 19 years and over.
Intra-aortic balloon pump (IABP) – used in sensitivity analysis	2776	NICE Medical technology guidance 8.[11] Costs have been inflated using the HCHS inflation index.
Intravenous fluids (Gelatin, 1500ml)	13.49	BNF[4]
Insulin (1000 units for 5 days)	138.60	BNF[4]

Treatment/Action	Unit cost+ (£)	Reference
Intravenous heparin (initial 5,000 units, then 15,000 units every 12 hours for 5 days)	47.43	BNF[4]
Isolation room, stool culture and IV for dehydration	3592	NHS Reference Costs 2012/13[1]
Omeprazole (IV omeprazole 40mg for 3 days, then 40mg oral daily for 5 days)	12.68	BNF,[4] eMIT[8]
Laparoscopy	2693	As laparotomy
Laparotomy	2693	NHS Reference Costs 2012/13[1]
Laxatives, enemas (Bisacodyl, 5mg; Sodium citrate, assume for 5 days)	2.25	BNF,[4] eMIT[8]
Minor treatment for wound dehiscence	161	NHS Reference Costs 2012/13.[1] Elective inpatients. JC43A Minor Skin Procedures, 13 years and over. 320 Cardiology, with the costs associated with the average LOS reported subtracted at a cost of £265 per day.
MRI scan	248	NHS Reference Costs 2012/13.[1] Diagnostic Imaging - Direct Access. RA07Z Magnetic Resonance Imaging Scan, requiring extensive patient repositioning and/or more than one contrast agent. 320 Cardiology
Nasendoscopy	115	As fluoroscopy
Nasogastric tube insertion	252	NHS Reference Costs 2012/13[1]
Negative Pressure Wound Therapy (NPWT) therapy	3501	NHS Reference Costs 2012/13.[1] Elective inpatients. JC42A Intermediate Skin Procedures, 13 years and over. 172 Cardiac Surgery, with the costs associated with the average LOS reported subtracted at a cost of £265 per day.
Nubulised 0.9% saline (10ml) or salbutamol (2.5 mg) 4 times daily	1035	NHS Reference Costs 2012/13.[1]
Parenteral nutrition (assume 5 days)	200	NICE, Nutrition support in adults: oral nutrition support, enteral tube feeding and parenteral nutrition costing report.[12] Costs have been inflated using the HCHS

Treatment/Action	Unit cost+ (£)	Reference
		inflation index.
Permanent pacemaker	14564	NHS Reference Costs 2012/13[1]
Physiotherapy/rehabilitation	139	NHS Reference Costs 2012/13.[1] Elective inpatients. DZ30Z Chest Physiotherapy. 340 Respiratory Medicine.
Speech and language therapy review	109	NHS Reference Costs 2012/13.[1] Non Consultant Led Outpatient Attendances. WF01B Non-Admitted Face to Face Attendance, First. 652 Speech and Language Therapy.
Stroke (alternative cost used in sensitivity analysis)	705	NHS Reference Costs 2012/13.[1] Non elective inpatients. AA35E Stroke with CC Score 4-6, 300 General Medicine, with the costs associated with the average LOS reported subtracted at a cost of £265 per day.
Temporary pacemaker	3073	NHS Reference Costs 2012/13.[1] Elective inpatients. EA39B Pacemaker Procedure without Generator Implant, including Re-siting and Removal of Cardiac Pacemaker System, with CC Score 2-4, 320 Cardiology, with the costs associated with the average LOS reported subtracted at a cost of £265 per day.
Tracheostomy	5313	NHS Reference Costs 2012/13.[1]
Ultrasound	67	NHS Reference Costs 2012/13.[1] Diagnostic Imaging – Outpatients. Weighted average of RA25Z Ultrasound Mobile Scan or Intraoperative Procedures, less than 20 minutes; RA26Z Ultrasound Mobile Scan or Intraoperative Procedures, 20 to 40 minutes, RA27Z Ultrasound Mobile Scan or Intraoperative Procedures, more than 40 minutes. 100 General Surgery.

BIPAP, Bilevel positive airway pressure; BNF, British National Formulary; CPAP, Continuous positive airway pressure; CPR, Cardiopulmonary resuscitation; CT, Computed tomography; ECG, Electrocardiography; eMIT, electronic Marketing Information Tool; HCHS, Hospital and community health services; IV, Intravenous; LOS, Length of stay; MRI, Magnetic resonance imaging; NICE, National Institute for Health and Care Excellence

Table 7 Unit costs for reattending hospital

Resource	Unit cost (£)	Reference
Ward day for readmissions	265	NHS Reference Costs 2012/13.[1] Non-elective inpatient excess bed day cost across all activities.
ICU day for readmissions	1168	NHS Reference Costs 2012/13.[1] Critical Care Services - Adult: Critical Care Unit (weighted average of XC01Z - XC07Z, 0-6 organs supported).
A&E attendance, leading to admission	154	NHS Reference Costs 2012/13.[1] Accident and Emergency Services, excluding dental care. Weighted average of all admitted codes.
A&E attendance, not leading to admission	101	NHS Reference Costs 2012/13.[1] Accident and Emergency Services, excluding dental care. Weighted average of all non-admitted codes.
Ambulance to hospital	230	NHS Reference Costs 2012/13.[1] Ambulance services, ASS02, See and treat and convey.

A&E, Accident and Emergency; ICU, Intensive care unit

Table 8 Resource use assumed for readmission complications and total costs

Complication	Treatment/Action	Cost (£)	Assumption
Antibiotics			
- Site=respiratory	Antibiotics, chest x-ray	69.40	
- Site=surgical wound	Antibiotics, chest x-ray, CT scan	131.40	
- Site=blood	Antibiotics	28.40	
- Site=other - endocarditis	Antibiotics, chest x-ray, CT scan	131.40	
- Site=other - infective endocarditis	Antibiotics, chest x-ray, CT scan, transthoracic echo	252.40	
- Site=other - respiratory tract infection	Antibiotics, chest x-ray	69.40	
- Site=other - wound	Antibiotics, chest x-ray, CT scan	131.40	
- Site=other - all others including UTI	Antibiotics	28.40	

Complication	Treatment/Action	Cost (£)	Assumption
Deep vein thrombosis	Duplex scan of leg veins, intravenous heparin, warfarin	203.78	
Cardiac tamponade	Transoesophageal echo, chest x-ray, 2 RBCs	559.62	Reoperations captured elsewhere
Other GI complications			
Barrett's oesophagus	Endoscopy	676	
Dehydration, hypovolaemia secondary to 3 days of diarrhoea	Intravenous fluids	13.49	
Gastroenteritis	Intravenous fluids	13.49	
Oesophageal ulcer	Endoscopy	676	
Peritonitis	Laparotomy, CT scan	2755	
Vomiting related to amiodarone. Medication changed.	Intravenous fluids	13.49	
Other pulmonary complications – all assumed to have 2 chest x-rays and an ECG – add £135 to all			
Acute shortness of breath - treated with diuretics	Diuretics, transthoracic echo	121.15	
Breathing difficulties at routine outpatients. Kept in overnight for breathing assessment	Transthoracic echo	121	
Breathlessness and cough	Transthoracic echo	121	
Chest pain and shortness of breath	Transthoracic echo	121	
Chest pain on inspiration/coughing	CT scan	62	
Cough	No treatment	0	
Dyspnoea	Transthoracic echo	121	
End stage heart failure	Diuretics, transthoracic echo	121.15	
Failed extubation	No additional treatment	0	Covered in ICU cost
Left hydropneumothorax	Chest ultrasound, chest drain	4244	
Musculoskeletal chest pain	Analgesia	6	

Complication	Treatment/Action	Cost (£)	Assumption
Pleural effusion and bilateral pedal oedema	Chest ultrasound, diuretic therapy	67.15	
Pleural effusion not requiring drainage	Chest ultrasound	67	
Pleural effusion treated with increased dose of furosemide	Furosemide (80mg)	0.30	
Pleuritic left lung (not requiring drainage)	Analgesia	6	
Pulmonary fibrosis	No treatment	0	
Right sided pleural effusion and empyema, ultrasound guided drainage, 2 units blood transfused, treated with IV antibiotics	Chest drain, 2 units RBCs, IV antibiotics	4452.02	
Severe chest pain, possible PE but ruled out following investigations	Transthoracic echo, CT pulmonary angiogram (CT chest)	183	
Shortness of breath	Transthoracic echo	121	
Shortness of breath due to fluid overload, diuretics increased	Transthoracic echo, furosemide (80mg)	121.30	
Sudden onset of shortness of breath, small right pleural effusion	Transthoracic echo	121	
Suspected PE - diagnosed with pleuritic chest pain	CT scan, transthoracic echo	183	
Other arrhythmia complications – all assumed to have 2 ECGs, add £106 to all			
Accelerated junctional rhythm	No additional treatment	0	Pacing already captured
Atrial fibrillation	Amiodarone	4.79	
Chest discomfort, palpitations	No treatment	0	
Fast atrial flutter	Amiodarone	4.79	
Paroxysmal AF	Amiodarone	4.79	
Re-entry tachycardia	No additional treatment	0	Only cost permanent pacemaker if

Complication	Treatment/Action	Cost (£)	Assumption
			clear indication participant had this treatment
Other thromboembolic complications			
Pulmonary embolus	Transthoracic echo, CT pulmonary angiogram (CT chest), intravenous heparin for 5 days, warfarin	231.78	
Apical thrombus	Transthoracic echo, intravenous heparin for 5 days, warfarin	169.78	
Possible bilateral renal infarcts	CT scan, intravenous heparin, warfarin	110.78	
Small leg thrombus	Ultrasound of leg, intravenous heparin, warfarin	115.78	

AF, Atrial fibrillation; CT, Computed tomography; ECG, Electrocardiography; GI, Gastrointestinal; ICU, Intensive care unit; IV, Intravenous; PE, Pulmonary embolism; RBC, Red blood cell; UTI, Urinary tract infection

Notes:

Resource use assumed for readmission complications and total costs are shown here, unit costs and sources are shown in the next table. Resource use and costs for readmission complications as inpatient complications if not reported here.

Table 9 Unit costs for readmission complications (not previously presented)

Treatment/Action	Unit cost (£)	Reference
Diuretics (furosemide 80mg orally for 5 days)	0.30	BNF[4]
Warfarin (3mg daily, assumed given for half of follow up time, 45 days)	1.35	BNF[4]

BNF, British National Formulary

Table 10 Unit costs for outpatient appointments

Speciality	Unit cost (£)	Service code	Reference
Anticoagulation service	25	324	These are all sourced from NHS Reference Costs 2012-13.[1] They are all average costs for each speciality (from the Total - Outpatient Attendances page, Total activity).
Cardiac rehabilitation	42	327	
Cardiac surgery	299	172	
Cardiology	131	320	
Cardiothoracic surgery	275	170	
Clinical haematology	151	303	
Colorectal surgery	113	104	
Dermatology	98	330	
Diabetic medicine	136	307	
Endocrinology	152	302	
Gastroenterology	137	301	
General medicine	153	300	
General surgery	128	100	
Geriatric medicine	204	430	
Hepatology	213	306	
Infectious diseases	142	350	
Medical oncology	138	370	

Speciality	Unit cost (£)	Service code	Reference
Nephrology	158	361	
Neurology	176	400	
Occupational therapy	63	651	
Ophthalmology	86	130	
Physiotherapy	42	650	
Plastic surgery	88	160	
Rehabilitation	90	314	
Respiratory medicine	150	340	
Stroke clinic	200	328	
Thoracic surgery	253	173	
Upper gastrointestinal surgery	120	106	
Urology	101	101	
Vascular surgery	142	107	

Table 11 Unit costs for other outpatient attendances

Resource	Unit cost (£)	Reference
Renal/ dialysis	157	NHS Reference Costs 2012/13.[1] Renal Dialysis at Base. LD02A Hospital Haemodialysis or Filtration, with Access via Arteriovenous Fistula or Graft, 19 years and over.
Outpatient endoscopy	676	As endoscopy, previously given
ECG	53	As previously given
Electrocardiogram monitoring and stress testing	204	As previously given
CT scan	62	As previously given
Echo scan - transthoracic	121	As previously given
MRI scan	248	As previously given
Chest x-ray	41	As previously given
Chest x-ray and ultrasound	108	As previously given
Sigmoidoscopy	164	NHS Reference Costs 2012/13.[1] Procedures in Outpatients. FZ57Z Diagnostic or Therapeutic, Rigid Sigmoidoscopy, 19 years and over. 104 Colorectal Surgery.

CT, Computed tomography; ECG, Electrocardiography; MRI, Magnetic resonance imaging

Table 12 Costs for resource use associated with serious adverse events (not previously presented)

Treatment/Action	Unit cost+ (£)	Reference
Bladder cystoscopy	129	NHS Reference Costs 2012/13.[1] Procedures in Outpatients. LB15E Minor Bladder Procedures, 19 years and over. 101 Urology.
Colonoscopy	257	NHS Reference Costs 2012/13.[1] Procedures in Outpatients. FZ51Z Diagnostic Colonoscopy, 19 years and over. 100 General Surgery
Diverticulitis	686	NHS Reference Costs 2012/13.[1] Elective inpatients. FZ83H Major Oesophageal, Stomach or Duodenum Procedures, 19 years and over with CC Score 4-6. 301 Gastroenterology, with the costs associated with the average LOS reported subtracted at a cost of £265 per day.
ECG	477	NHS Reference Costs 2012/13.[1] Day Cases. EA47Z Electrocardiogram Monitoring and stress testing. 320 Cardiology.
Fasciotomy	6182	NHS Reference Costs 2012/13.[1] Non-elective inpatients. QZ02D Lower Limb Arterial Surgery with CC Score 6-10. 107 Vascular Surgery, with the costs associated with the average LOS reported subtracted at a cost of £265 per day.
Gastroscopy	676	As endoscopy
Groin scan/procedure of lymphatic system	3191	NHS Reference Costs 2012/13.[1]
Oesophagogastroduodenoscopy	676	As endoscopy
Leg amputation	13353	NHS Reference Costs 2012/13.[1] Non-elective inpatients. QZ11D Amputations with CC Score 8-13. 107 Vascular Surgery.
Re-catherisation	1534	NHS Reference Costs 2012/13[1]
Stoma bag system	63.38	NHS Electronic Drug Tariff.[13] Part IXC - Stoma Appliances (Colostomy Sets). Weighted average of all sets.
Tesio-catheter insertion under fluoroscopy	325	NHS Reference Costs 2012/13[1]
Uroscopy	129	NHS Reference Costs 2012/13.[1] Procedures in Outpatients. LB15E Minor Bladder

Treatment/Action	Unit cost+ (£)	Reference
		Procedures, 19 years and over. 101 Urology.

ECG, Electrocardiography

Table 13 Unit costs for post-discharge community health and social care contacts

Resource	Unit cost+ (£)	Reference
GP at surgery	34	Unit Costs of Health and Social Care 2013.[14] 10.8b, GP - unit costs. Per patient contact lasting 11.7 minutes. Excluding qualification costs and direct care staff costs.
GP at home	85	Unit Costs of Health and Social Care 2013.[14] 10.8b, GP - unit costs. Per out of surgery visit lasting 23.4 minutes. Excluding qualification costs and direct care staff costs.
Out-of-hours GP	34	As GP at surgery
Walk-in centre	34	As GP at surgery
GP nurse	11.37	Unit Costs of Health and Social Care 2013.[14] 10.6, Nurse (GP practice). £44 per hour of face-to-face contact, excluding qualification costs. Average contact 15.5 minutes.
District nurse	39	Unit Costs of Health and Social Care 2013.[14] 10.1, Community Nurse. Using data from NHS Reference Costs 2011/12,[1] the mean average cost for a face-to-face contact in district nursing services for 2012/2013 was £39, with an interquartile range of £33 to £46. Costs have been uprated using the HCHS pay & prices inflator.
**Other NHS or social services**		
Cardiac rehabilitation / exercise class	42	NHS Reference Costs 2012/13.[1] Total - Outpatient Attendances. 327 - Cardiac Rehabilitation.
Cardiac nurse	70	NHS Reference Costs 2012/13.[1] Community Health Services – Nursing, N11AF, Specialist Nursing - Cardiac Nursing/Liaison, Adult, Face to face.

Resource	Unit cost[+] (£)	Reference
Diabetic nurse	70	NHS Reference Costs 2012/13.[1] Community Health Services – Nursing, N15AF, Specialist Nursing - Diabetic Nursing/Liaison, Adult, Face to face.
Anticoagulation service	10	NHS Reference Costs 2012/13.[1] Non Consultant Led Outpatient Attendances; Non-Admitted Non-Face to Face Attendance, Follow-up, 324 - Anticoagulation Service.
Community pharmacist	69.64	Unit Costs of Health and Social Care 2013.[14] 9.6, Community pharmacist. £127 per hour of direct clinical activities. Contact assumed to be for 32.9 minutes [a].
Cardiac rehabilitation by phone	50	NHS Reference Costs 2012/13.[1] Non Consultant Led Outpatient Attendances; Non-Admitted Non-Face to Face Attendance, Follow-up, 327 - Cardiac Rehabilitation.
Dietician	71	NHS Reference Costs 2012/13.[1] Community Health Services - Allied Health Professionals. A03 – Dietician.
Health care support worker	16	Unit Costs of Health and Social Care 2013.[14] 10.5, Clinical support worker nursing (community). £30 per hour of home visiting. Contact assumed to be for 32.9 minutes [a].
Occupational therapist	53	Unit Costs of Health and Social Care 2013.[14] 13.2, Hospital occupational therapist. Using data from NHS Reference Costs 2011/12, the mean average cost for a non-consultant led (non-admitted) follow-up occupational therapy attendance was £53, with an interquartile range of £30 to £64. Costs have been uprated using the HCHS pay & prices inflator.
Physiotherapist	34	Unit Costs of Health and Social Care 2013.[14] 13.1, Hospital physiotherapist. Using data from NHS Reference Costs 2011/12, the mean average cost for a non-consultant-led (non-admitted) follow-up physiotherapy attendance was £34, with an interquartile range of £28 to £38. Costs have been uprated using the HCHS pay & prices inflator.
Social worker	87.19	Unit Costs of Health and Social Care 2013.[14] 11.2, Social worker (adult services). £159 per hour of face-to-face contact, excluding qualification costs. Contact assumed to be for 32.9 minutes [a].
**Other NHS or social services at home**		
Cardiac rehabilitation /	70	NHS Reference Costs 2012/13.[1] Community Health Services – Nursing. N11AF Specialist Nursing - Cardiac

Resource	Unit cost[+] (£)	Reference
nurse		Nursing/Liaison, Adult, Face to face.
Phone call to cardiology	49	NHS Reference Costs 2012/13.[1] Outpatients. WF01C, Non-Admitted Non-Face to Face Attendance, Follow-up. 320 – Cardiology.
Carer	8.50	Unit Costs of Health and Social Care 2013.[14] 11.6, Home care worker. The mean hourly cost of all home care including LA-funded and independent provision was £17. Just over half of local authority funded visits lasted 30 minutes. Sixteen per cent of visits were 15 minutes and 19 per cent of a home care workers' time was spent travelling. Assume 30 minute visit.
Community matron	68	NHS Reference Costs 2012/13.[1] Community Health Services – Nursing. N06AF - Specialist Nursing - Active Case Management (Community Matrons), Adult, Face to face.
Community mental health team carer	36	Unit Costs of Health and Social Care 2013.[14] 10.2 Nurse (mental health), £65 per hour of face-to-face contact, excluding qualifications. Contact assumed to be for 32.9 minutes [a].
Dietician	71	NHS Reference Costs 2012/13.[1] Community Health Services - Allied Health Professionals. A03 – Dietician.
Occupational therapist	73	Unit Costs of Health and Social Care 2013.[14] 9.2, NHS community occupational therapist. Using data from NHS Reference Costs 2011/12, the mean average cost for a one-to-one contact of occupational therapy services was £73, with an interquartile range of £50 to £86. Costs have been updated using the HCHS pay & prices inflator.
Paramedic	174	NHS Reference Costs 2012/13.[1] Ambulance Services. ASS01-See and treat or refer.
Physiotherapist	47	Unit Costs of Health and Social Care 2013.[14] 9.1, Community physiotherapist. Using data from NHS Reference Costs 2011/12, the mean average cost for a one-to-one contact in physiotherapy services was £47, with an interquartile range of £37 to £52. Costs have been uprated using the HCHS pay & prices inflator.
Social worker	87.19	Unit Costs of Health and Social Care 2013.[14] 11.2, Social worker (adult services). £159 per hour of face-to-face contact, excluding qualification costs. Contact assumed to be for 32.9 minutes [a].
Nurse specialist	60	NHS Reference Costs 2012/13.[1] Community Health Services – Nursing, N29AF, Other Specialist Nursing, Adult, Face to face.

Resource	Unit cost[+] (£)	Reference
Respiratory nurse	75	NHS Reference Costs 2012/13.[1] Community Health Services – Nursing, N08AF, Specialist Nursing - Asthma and Respiratory Nursing/Liaison, Adult, Face to face.
NHS direct call	13	NHS Direct annual report 2012/13.[15]
Indoor and outdoor grab rails	91	Unit Costs of Health and Social Care 2013.[14] 7.3.1, social services access improvements.
Mobile shower chair	55	Unit Costs of Health and Social Care 2013.[14] 7.3.1, social services shower.

GP, General practitioner; HCHS, Hospital and community health services

Notes for supplementary tables:

[+]Unit costs which were not in 2012/2013 prices were inflated to 2012/2013 prices using the hospital and community health services (HCHS) inflation index.[14]

[a] Where no information was available on the duration of appointments, an average duration of 32.9 minutes has been assumed. This is the average length of a hospital physiotherapy session (Unit Costs of Health and Social Care 2013;[14] section 13.1).

Appendix 3. Stokes et al. TITRe2 Cost-effectiveness paper

Sensitivity analyses

Sensitivity analyses for costing were conducted to investigate varying a number of unit costs, moving the time origin from surgery to the time of randomisation, and the impact of any high cost participants. In terms of outcomes, alternative assumptions for calculating QALYs were implemented in sensitivity analyses. Finally we examined life-years gained as a secondary outcome measure. Each of these sensitivity analyses is considered in turn.

Sensitivity analyses around unit costs

The sensitivity analyses conducted around the costs of bed days, antibiotics, complications and outpatient visits and results are shown in Tables 14 and 15. Varying the costs of bed days during the index admission by +/- 50% had the greatest impact on total costs in each group (increasing and decreasing total costs to approximately £21,000 and £15,000 respectively). However, none of the sensitivity analyses had a great impact on the cost difference between the groups. The cost differences across the sensitivity analyses ranged from -£208 to -£161, bracketing and all very similar to the base case cost difference of -£182. These findings reinforce how similar resource use is between the groups.

Table 14 Sensitivity analyses performed around unit costs

SA	Resource / complication	Unit costs used in base case analysis	Alternative strategies for sensitivity analysis
1	Ward stay in cardiac unit (first admission)	£392	£265 (Cost used for ward stay beyond index cardiac admission)
2	Ward stay beyond index cardiac admission. (Further stay in another unit/hospital or readmission)	£265	£392 (Cost used for ward stay in cardiac unit, first admission)
3	Bed days in first admission	£1,608 General ICU<br>£1,190 CICU<br>£619 HDU<br>£392 cardiac ward<br>£265 another unit/hospital ward	Alter bed day costs in first admission by +/- 25% and 50%

		(£1,168 if known to be ICU)	
4	Bed days in readmissions	£1,168 ICU<br>£265 ward	Alter readmission ICU / ward costs by +/- 25% and 50%
5	Stroke	£139 for physiotherapy, and diagnostics as recorded (CT scan £62; MRI scan £248)	£705 (taken from Reference Costs,[1] see Table 6)
6	Wound dehiscence	Covered by reoperation if the 2 dates are the same; otherwise £161 unless negative pressure wound therapy is stated then £3501	Assume negative pressure wound therapy for those without reoperations (£3501)
7	Low cardiac output	£313; IABP not included	Add cost of IABP £2776
8	Chest drain	£4177	-50%: £2088.50
9	Pacing	£3073	+/- 25% and 50%
10	Tracheostomy	£5354 (includes 1 x-ray)	+/- 25% and 50%
11	Reoperations	£6608 if operation takes less than 3 hours, and £8298 if 3 hours or more. Reoperations in readmissions costed at £6608 + £1421 for blood products.	Cost all reoperations at the lower (£6608) and higher figures (£8298). Include £1421 for blood products for reoperations in readmissions.
12	Antibiotics	eMIT[8] where available, otherwise BNF;[4] see Table 3	Increase costs by 100%
13	Antibiotics	eMIT[8] where available, otherwise BNF;[4] see Table 3	Cost most common antibiotics (those received by 20 or more participants) using BNF;[4] see Table 3
14	Antibiotics	eMIT[8] where available, otherwise BNF;[4] see Table 3	For antibiotics participants receive orally or intravenously, cost all as oral
15	Antibiotics	eMIT[8] where available, otherwise BNF;[4] see Table 3	For antibiotics participants receive orally or intravenously, cost all as intravenous
16	Outpatient visits	See Table 10 and Table 11	+/- 25% and 50%

BNF, British National Formulary; CICU, Cardiac intensive care unit; CT, Computed tomography; eMIT, electronic Marketing Information Tool; HDU, High dependency unit; IABP, Intra-aortic balloon pump; ICU, Intensive care unit; MRI, Magnetic resonance imaging; SA, Sensitivity analysis

Table 15 Results of sensitivity analyses around unit costs

Sensitivity Analysis	Randomised to restrictive threshold (N=1000) Mean cost (£) (SE)	Randomised to liberal threshold (N=1003) Mean cost (£) (SE)	Restrictive versus liberal threshold Mean cost (£) difference (SE)
**Base case**	**17945 (332)**	**18127 (357)**	**-182 (488)**
SA1 (Ward stay, cardiac unit)	17226 (327)	17386 (352)	-161 (480)
SA2 (Ward stay, beyond index cardiac admission)	18267 (346)	18476 (370)	-208 (507)
SA3 (Bed days, first admission)			
+25%	19409 (377)	19600 (408)	-191 (556)
-25%	16482 (289)	16654 (308)	-173 (422)
+50%	20872 (423)	21073 (460)	-201 (625)
-50%	15018 (248)	15181 (261)	-163 (360)
SA4 (Bed days, readmissions)			
+25%	18057 (336)	18239 (360)	-182 (493)
-25%	17834 (329)	18016 (355)	-182 (484)
+50%	18168 (341)	18350 (363)	-182 (498)
-50%	17722 (326)	17904 (353)	-182 (481)
SA5 (Stroke)	17954 (333)	18137 (358)	-182 (489)
SA6 (Wound dehiscence)	18022 (337)	18200 (361)	-178 (494)
SA7 (Low cardiac output)	18248 (341)	18439 (369)	-192 (502)
SA8 (Chest drain)	17712 (324)	17905 (348)	-193 (475)
SA9 (Pacing)			
+25%	18182 (336)	18366 (361)	-184 (493)
-25%	17709 (329)	17888 (355)	-180 (484)
+50%	18419 (340)	18605 (364)	-187 (498)
-50%	17472 (326)	17649 (352)	-177 (480)
SA10 (Tracheostomy)			
+25%	17991 (337)	18171 (362)	-180 (495)
-25%	17900 (327)	18083 (352)	-183 (481)
+50%	18036 (342)	18215 (367)	-179 (502)
-50%	17854 (323)	18039 (348)	-185 (474)
SA11 (Reoperations)			
£6608	17930 (331)	18115 (356)	-185 (486)
£8298	18092 (339)	18296 (366)	-203 (499)
SA12 (Antibiotics)	17968 (334)	18143 (358)	-175 (490)
SA13 (Antibiotics, BNF)	18004 (335)	18190 (361)	-186 (492)

SA14 (Antibiotics, oral)	17943 (332)	18126 (357)	-182 (488)
SA15 (Antibiotics, IV)	17948 (332)	18131 (358)	-183 (488)
SA16 (Outpatient visits)			
+25%	17995 (332)	18181 (357)	-185 (488)
-25%	17895 (332)	18074 (357)	-178 (488)
+50%	18045 (332)	18234 (357)	-189 (488)
-50%	17845 (333)	18020 (358)	-175 (488)

BNF, British National Formulary; IV, Intravenous; SA, Sensitivity analysis; SE, Standard error

Costing from the point of randomisation

Events that occurred before randomisation were excluded and costs from randomisation to 3 months calculated. Participants were on average randomised 0.8 days after surgery. Tables 16 and 17 present the mean resource use and mean costs to 3 months from randomisation. Participants in the restrictive group received on average 1 less unit of red blood cells than participants in the liberal group; other resource use was similar between the groups. There is little difference in total costs of care from randomisation to 3 months between the two treatment groups. The total costs from randomisation are £8,825 (SE 310) in the restrictive group, and £8,959 (SE 340) in the liberal group, with a mean difference between the groups of -£134 (SE 460). The costs associated with red blood cells are lower in the restrictive group compared to the liberal group as expected.

Total costs from randomisation are considerably less than total costs from surgery. Costs are lower because the costs of surgery and complications occurring before randomisation have been excluded and LOS costs are reduced. The LOS occurring pre-randomisation is at least in part time spent in CICU/HDU, since all participants go to CICU/HDU after surgery. Red blood cell costs are also reduced, since red blood cells are sometimes transfused during surgery.

Table 16 Resource use per participant to 3 months from randomisation

Resource use component	Randomised to restrictive threshold (N=1000) Frequency (%) or Mean (SE)	Randomised to liberal threshold (N=1003) Frequency (%) or Mean (SE)	Restrictive versus liberal threshold % or Mean (SE) Difference
**Red blood cells** – number of units/participant	1.49 (0.08)	2.49 (0.09)	-1.00 (0.12)
**Blood products** – number of			
units/participant	1.00 (0.06)	0.95 (0.06)	0.05 (0.08)
Fresh frozen plasma	0.65 (0.03)	0.64 (0.03)	0.01 (0.05)
Platelets	0.23 (0.03)	0.21 (0.02)	0.02 (0.04)
Cryoprecipitate			
**Inpatient complications**			
**Primary outcome** - number (%) of participants			
Antibiotics for infectious complication	319 (32%)	322 (32%)	0%
Stroke	11 (1%)	14 (1%)	0%
Suspected myocardial infarction	2 (0%)	4 (0%)	0%
Gut infarction	5 (1%)	1 (0%)	0%
Acute kidney injury - stage 3	47 (5%)	45 (4%)	0%
**Other complications** – number of events/participant			
Reoperation	0.07 (0.01)	0.08 (0.01)	-0.01 (0.01)
Reintubation	0.05 (0.01)	0.06 (0.01)	-0.01 (0.01)
Trachestomy	0.03 (0.01)	0.03 (0.01)	0.00 (0.01)
Mask CPAP	0.10 (0.01)	0.09 (0.01)	0.01 (0.02)
Pneumothorax requiring chest drainage	0.01 (0.00)	0.01 (0.00)	0.00 (0.00)
Pleural effusion requiring drainage	0.06 (0.01)	0.06 (0.01)	0.00 (0.01)
Pacing	0.09 (0.01)	0.06 (0.01)	0.03 (0.01)
SVT/AF requiring treatment	0.35 (0.02)	0.33 (0.02)	0.02 (0.03)
VF/VT requiring intervention	0.02 (0.01)	0.01 (0.00)	0.01 (0.01)
Low cardiac output	0.03 (0.01)	0.03 (0.01)	0.01 (0.01)
**Inpatient length of stay** - days/participant			
Cardiac intensive care unit	0.89 (0.11)	0.86 (0.13)	0.03 (0.17)
High dependency unit	2.75 (0.12)	2.71 (0.12)	0.04 (0.17)
Ward	5.49 (0.15)	5.68 (0.17)	-0.19 (0.22)
Another unit / hospital	1.26 (0.18)	1.36 (0.21)	-0.09 (0.28)

**Blood saving techniques** – number (%) of participants			
Post-operative cell salvage*	24 (2%)	20 (2%)	0%
**Readmissions to hospital**			
Length of stay – days/participant	1.39 (0.15)	1.48 (0.16)	-0.09 (0.22)
**A&E attendances**			
Total A&E visits - number/participant	0.08 (0.01)	0.07 (0.01)	0.01 (0.01)
**Outpatient appointments** - number/participant			
Cardiac surgery outpatient visits	0.44 (0.02)	0.51 (0.02)	-0.07 (0.03)
Cardiology outpatient visits	0.28 (0.02)	0.26 (0.02)	0.02 (0.03)
Other outpatient visits	0.17 (0.02)	0.17 (0.02)	-0.01 (0.03)
**Other health care contacts** - number/participant			
General practitioner at surgery	1.99 (0.07)	2.10 (0.08)	-0.11 (0.10)
General practitioner at home	0.43 (0.05)	0.38 (0.03)	0.05 (0.06)
Practice nurse	1.55 (0.15)	1.57 (0.13)	-0.02 (0.18)
District nurse	2.40 (0.22)	2.18 (0.21)	0.22 (0.30)

A&E, Accident and Emergency; CPAP, Continuous positive airway pressure; SE, Standard error; SVT/AF, Supraventricular tachycardia/ Atrial fibrillation; VF/VT, Ventricular fibrillation/ Ventricular tachycardia

Notes:

** Included if participant randomised within 4 hours of surgery*

Table 17 Breakdown of total average costs per participant from randomisation to 3 months for both trial groups

Cost component	Randomised to restrictive threshold (N=1000) Mean cost (£) (SE)	Randomised to liberal threshold (N=1003) Mean cost (£) (SE)	Restrictive versus liberal threshold Mean cost (£) difference (SE)
**Red blood cells**	**208 (11)**	**349 (13)**	**-141 (17)**
**Inpatient episode**			
Other blood products	206 (12)	199 (11)	7 (16)
Complications and SAEs	1694 (120)	1663 (128)	31 (175)
Length of stay*	5274 (198)	5318 (219)	-45 (295)
Blood saving techniques	43 (8)	36 (7)	7 (10)
Regular medications	26 (2)	29 (2)	-3 (3)
**Total**	**7243 (286)**	**7245 (322)**	**-2 (430)**

**Post-discharge**			
Readmissions	780 (87)	765 (79)	15 (117)
A&E visits	16 (2)	12 (2)	4 (3)
Outpatient appointments	202 (6)	219 (7)	-17 (9)
Other medical/social care	376 (13)	369 (17)	7 (21)
**Total**	**1374 (92)**	**1365 (83)**	**9 (124)**
**Total costs**	**8825 (310)**	**8959 (340)**	**-134 (460)**

A&E, Accident and Emergency; SAE, Serious adverse event; SE, Standard error

Notes:

** Includes days in another unit/hospital once transferred out of the cardiac unit*

Sensitivity analyses around cost outliers

The results of this sensitivity analysis were described in the main text. Table 18 provides further details of the effects on costs and cost-effectiveness results of excluding the highest cost outlier, and of excluding the four highest cost outliers with total costs over £100,000. All of these participants were in the liberal group, so results for the restrictive group are unchanged. If the participant with the highest cost is excluded from the analyses, the difference in costs between the groups reduces from -£182 to -£55. If participants with the four highest costs are excluded, the liberal group becomes less expensive than the restrictive group, the difference in costs between the groups changing from -£182 to +£208. The liberal group also becomes marginally more effective than the restrictive group and the conclusions are reversed, i.e. the liberal group dominates the restrictive group as it is both less costly and more effective. While there is much uncertainty around these findings, these four participants are clearly exerting a significant impact on the cost and cost-effectiveness results.

Table 18 Sensitivity analyses around the cost outliers

	Randomised to restrictive threshold (N=1000)		Randomised to liberal threshold (N=1003)		Restrictive versus liberal threshold		
	**Mean costs (SE)**	**Mean QALYs (SE)**	**Mean costs (SE)**	**Mean QALYs (SE)**	**Mean cost difference (SE)**	**Mean QALY difference (SE)**	**ICER**
Base case - all participants	£17945 (£332)	0.1802 (0.0015)	£18127 (£357)	0.1798 (0.0016)	-£182 (£488)	0.0004 (0.0021)	Restrictive dominant (-£428,064)
Exclude highest cost participant	£17945 (£332)	0.1802 (0.0015)	£18001 (£335)	0.1799 (0.0016)	-£55 (£471)	0.0003 (0.0021)	Restrictive dominant (-£210,078)
Exclude 4 highest cost participants	£17945 (£332)	0.1802 (0.0015)	£17737 (£299)	0.1803 (0.0016)	£208 (£447)	-0.0001 (0.0021)	Liberal dominant (-£1,835,715)

ICER, Incremental cost-effectiveness ratio; QALY, Quality-adjusted life-year; SE, Standard error

Sensitivity analyses around outcomes

The various assumptions for calculating QALYs explored are described in Table 19, and the results are shown in Table 20. In all of these sensitivity analyses, the difference in QALYs between the groups remained very small. When last observation carried forward until death was used, QALYs increased slightly in both groups, more so in the restrictive group since there were more deaths in this group and a greater number of participants whose QALYs were increased by this sensitivity analysis (unless their EQ-5D score was less than zero at the previous observation). When the exact timing of the 6 week EQ-5D questionnaire was used in the QALY calculations, the mean QALYs gained to 3 months were slightly higher in the liberal group than in the restrictive group, a reversal of the base case findings. On average participants completed the 6 week questionnaire later than planned, at 51 days rather than 42 days.

Table 19 Sensitivity analyses performed around outcomes

SA	Aspect of methodology	Strategy used in base case analysis	Alternative strategy for sensitivity analysis
1	QALY calculations: adjusting for baseline utility	Regression used to adjust for differences in baseline utility	No adjustment for baseline utility
2	QALY calculations for participants who die	Utility was assumed to change linearly between the preceding time point and the time of death	Use last observation carried forward until death
3	QALY calculations: timing of 6 week EQ-5D	EQ-5D at 6 weeks assumed to be completed at exactly 6 weeks	Use the date of completion of the 6 week EQ-5D
4	QALY calculations: timing	Calculate QALYs gained from time of surgery to 3 months	Calculate QALYs gained from randomisation to 3 months

QALY, Quality-adjusted life-year; SA, Sensitivity analysis

Table 20 Results of sensitivity analyses around outcomes

Sensitivity Analysis		Randomised to restrictive threshold (N=1000) Mean (SE)	Randomised to liberal threshold (N=1003) Mean (SE)	Restrictive versus liberal threshold<br>Mean difference (SE)
	**QALYs to 3 months**			
	Base case	0.1802 (0.0015)	0.1798 (0.0016)	0.0004 (0.0021)
1	No adjustment for baseline utility	0.1801 (0.0018)	0.1798 (0.0017)	0.0003 (0.0025)
2	Last observation carried forward until death	0.1807 (0.0015)	0.1800 (0.0016)	0.0007 (0.0021)
3	Exact time between operation and 6 week EQ-5D completion	0.1801 (0.0014)	0.1802 (0.0014)	-0.0002 (0.0020)
4	From randomisation	0.1801 (0.0015)	0.1795 (0.0015)	0.0006 (0.0021)

QALY, Quality-adjusted life-year; SE, Standard error

Life-years as an alternative outcome measure

Life-years gained from surgery up to 3 months are shown in Table 21. Given the greater number of deaths in the restrictive group, slightly fewer life-years were gained in the restrictive group than in the liberal group. The cost-effectiveness results using life-years as the outcome measure are also shown in Table 21. This analysis generated the typical trade-off between effectiveness and cost; the restrictive threshold was less effective but also less costly than the liberal threshold. The ICER of £66,800 is the incremental saving associated with the loss of 1 life-year by adopting a restrictive rather than a liberal threshold. If a decision maker's willingness to accept compensation for the loss of one life-year were £20,000, then a restrictive threshold would be considered cost-effective.

The cost-effectiveness plane for life-years is presented in Figure 1; it shows quite interesting differences compared to the cost-effectiveness plane for the QALY analysis. In Figure 1 many of the points are located in the bottom left quadrant of the plane (south-west quadrant), indicating that a restrictive threshold is most likely to be less effective and less costly than a liberal threshold. With the QALYs analysis, the plane had most of its points scattered around the origin showing very little difference. Compared to the cost-effectiveness plane for QALYs, the points on the cost-effectiveness plane for life-years have been pulled across to the left, as there is a clearer difference in effects, namely a reduction in the number of life-years gained in the restrictive group; the uncertainty around the difference in costs remains. In this analysis, the probability that restrictive is more effective than liberal is just 3% (not statistically significant). There is only a 2% probability that the restrictive threshold dominates the liberal threshold (i.e. is more effective and less costly), but a 34% probability of the reverse scenario, that the liberal threshold dominates the restrictive threshold.

Table 21 Cost-effectiveness results for life-years

	**Total costs (95% CI)**			**Life-years gained (95% CI)**			**ICER**	**Probability that restrictive is**		**Probability restrictive is cost-effective at a ceiling ratio of**			**Probability that restrictive is**	
	Restrictive threshold (n=1000)	Liberal threshold (n=1003)	Restrictive versus liberal threshold	Restrictive threshold (n=1000)	Liberal threshold (n=1003)	Restrictive versus liberal threshold	Cost/life year	Dominant	Dominated	£20,000	£50,000	£100,000	More effective	Less costly
Life-years	£17945 (£17273, £18618)	£18127 (£17450, £18804)	-£182 (-£1108, £744)	0.2428 (0.2404, 0.2452)	0.2455 (0.2437, 0.2474)	-0.0027 (-0.0057, 0.0002)	£66,800	2%	34%	61%	55%	45%	3%	65%

CI, Confidence interval; ICER, Incremental cost-effectiveness ratio

Notes:

95% CI are based on parametric methods, using standard errors from the bootstrap replicates and a t distribution with degrees of freedom $v = (M-1)(1+r^{-1})^2$*, where r is the ratio of the between-imputation component of the variance and the within-imputation component of the variance and M is the number of imputed datasets.[16]*

Figure 1 Cost-effectiveness plane for life-years

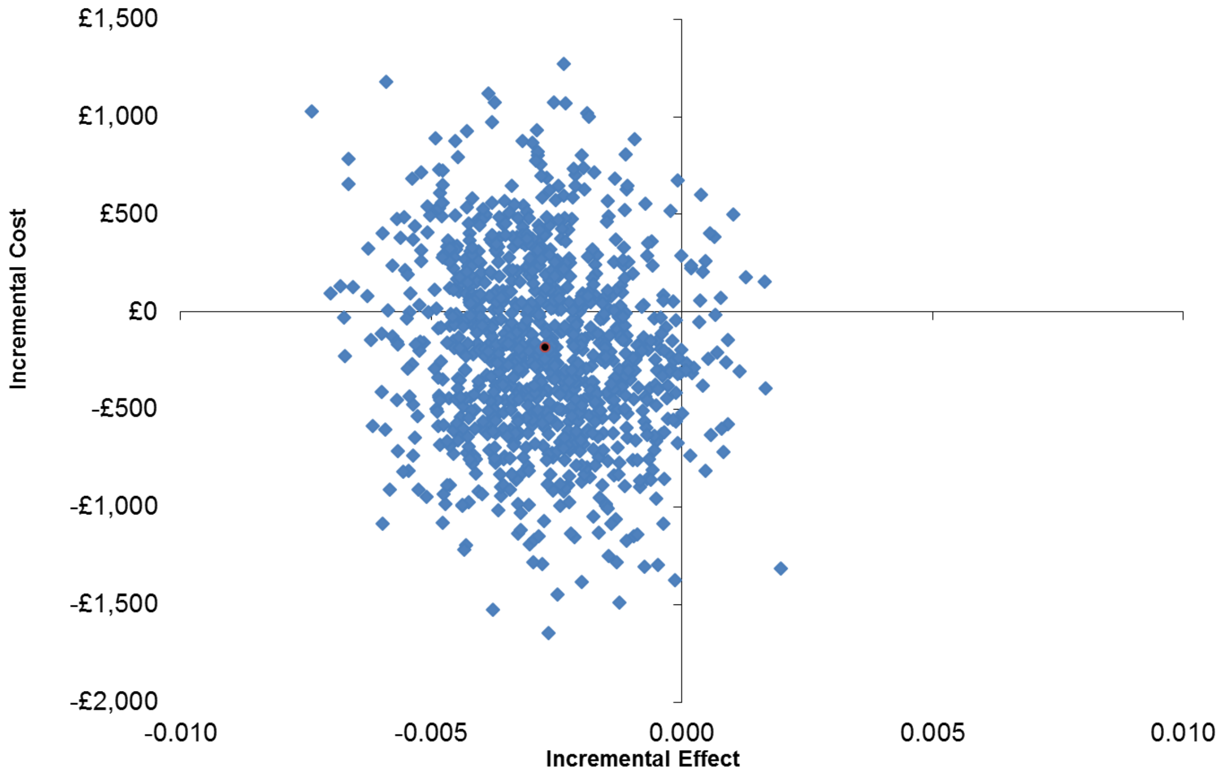

Appendix 4. Stokes et al. TITRe2 Cost-effectiveness paper

Subgroup analyses

The results of the seven subgroup analyses conducted to investigate whether cost-effectiveness results varied between participant subgroups are presented in Table 22. Comparing costs between subgroups as a whole, the findings are as expected: isolated CABG participants cost less than participants having other types of surgery; participants aged under 75 cost less than those aged over 75, and participants without each comorbidity before surgery cost less than those with the comorbidity (with the possible exception of pulmonary disease/asthma, but the numbers for this comorbidity were small).

The cost and QALY differences between the treatment groups within the subgroups are all small relative to their SEs. When the impact of subgroups was evaluated using ordinary least squares regression separately for total costs and for QALYs, and considering interaction terms between treatment group and subgroup, only the interaction term for the subgroup for lung disease for QALYs was found to be significant (p=0.003). Participants in the restrictive group with chronic pulmonary disease or asthma gained a reduced number of QALYs compared to other participants.

For subgroup analyses 1-4 and 6, the direction of differences between treatment groups does differ between the subgroups. In the “low risk” stratum of each subgroup analysis (participants believed to be at lower risk of the primary outcome): the restrictive threshold is both less costly and more effective than the liberal threshold and, therefore, a restrictive threshold is favoured. In the “high risk” stratum of each subgroup (participants believed to be at higher risk of the primary outcome): the restrictive threshold is both more costly and less effective than the liberal threshold, so the liberal threshold is favoured. Note that negative ICERs need to be interpreted with caution (an ICER is a ratio of two numbers and, if either of the two is negative, the ICER will be negative): either the new intervention is less costly and more effective, a desirable finding; or the new intervention is more costly and less effective, an undesirable finding; both scenarios result in negative ICERs, but have very different meanings. These subgroup analyses should be considered as exploratory, further work would be required to confirm these findings.

Table 22 Costs, QALYs and cost-effectiveness results for each of the subgroups

	"Low risk" stratum							"High risk" stratum						
	Restrictive		Liberal		Restrictive vs Liberal			Restrictive		Liberal		Restrictive vs Liberal		
	Mean costs (SE)	Mean QALYs (SE)	Mean costs (SE)	Mean QALYs (SE)	Mean cost difference (SE)	Mean QALY difference (SE)	ICER	Mean costs (SE)	Mean QALYs (SE)	Mean costs (SE)	Mean QALYs (SE)	Mean cost difference (SE)	Mean QALY difference (SE)	ICER
1	**Isolated CABG**							**Other operation types**						
	£14663<br>(£356)<br>n=408	0.1853<br>(0.0023)	£15218<br>(£406)<br>n=408	0.1819<br>(0.0022)	-£555<br>(£540)	0.0034<br>(0.0031)	Restrictive dominant<br>(-£161,423)	£20208<br>(£483)<br>n=592	0.1765<br>(0.0020)	£20122<br>(£519)<br>n=595	0.1784<br>(0.0021)	£86<br>(£709)	-0.0019<br>(0.0029)	Liberal dominant<br>(-£44,221)
2	**<75 years**							**≥75 years**						
	£17146<br>(£367)<br>n=714	0.1813<br>(0.0018)	£17290<br>(£437)<br>n=680	0.1796<br>(0.0019)	-£144<br>(£571)	0.0017<br>(0.0025)	Restrictive dominant<br>(-£86,221)	£19940<br>(£702)<br>n=286	0.1774<br>(0.0029)	£19889<br>(£609)<br>n=323	0.1800<br>(0.0029)	£51<br>(£929)	-0.0026<br>(0.0040)	Liberal dominant<br>(-£19,818)
3	**No diabetes**							**Diet, oral medication or insulin controlled diabetes**						
	£17365<br>(£352)<br>n=802	0.1827<br>(0.0016)	£17701<br>(£363)<br>n=802	0.1815<br>(0.0018)	-£336<br>(£506)	0.0012<br>(0.0024)	Restrictive dominant<br>(-£277,188)	£20296<br>(£869)<br>n=198	0.1699<br>(0.0039)	£19826<br>(£1035)<br>n=201	0.1729<br>(0.0039)	£469<br>(£1351)	-0.0029<br>(0.0056)	Liberal dominant<br>(-£160,777)
4	**No lung disease***							**Chronic pulmonary disease or asthma***						
	£17648	0.1833	£18150	0.1806	-£502	0.0028	Restrictive	£20325	0.1564	£17987	0.1736	£2338	-0.0172	Liberal

	(£330)<br>n=889	(0.0016)	(£399)<br>n=861	(0.0017)	(£518)	(0.0022)	dominant<br>(-£181,346)	(£1389)<br>n=111	(0.0048)	(£715)<br>n=142	(0.0041)	(£1562)	(0.0064)	dominant<br>(-£135,981)
5	**eGFR >60ml/min**							**eGFR ≤60ml/min**						
	£17342<br>(£381)<br>n=715	0.1820<br>(0.0017)	£17293<br>(£400)<br>n=698	0.1824<br>(0.0018)	£48<br>(£552)	-0.0004<br>(0.0024)	Liberal<br>dominant<br>(-£117,537)	£19460<br>(£661)<br>n=285	0.1757<br>(0.0031)	£20077<br>(£729)<br>n=303	0.1735<br>(0.0032)	-£617<br>(£984)	0.0022<br>(0.0044)	Restrictive<br>dominant<br>(-£275,536)
6	**Males**							**Females**						
	£17982<br>(£421)<br>n=693	0.1836<br>(0.0019)	£18367<br>(£468)<br>n=680	0.1823<br>(0.0020)	-£386<br>(£629)	0.0012<br>(0.0026)	Restrictive<br>dominant<br>(-£314,941)	£17863<br>(£519)<br>n=307	0.1728<br>(0.0026)	£17622<br>(£511)<br>n=323	0.1741<br>(0.0028)	£241<br>(£728)	-0.0013<br>(0.0039)	Liberal<br>dominant<br>(-£183,713)
7	**Good ventricular function**							**Moderate or poor ventricular function**						
	£17667<br>(£371)<br>n=787	0.1831<br>(0.0016)	£17582<br>(£404)<br>n=771	0.1827<br>(0.0017)	£85<br>(£549)	0.0004<br>(0.0022)	£210,032	£18854<br>(£706)<br>n=204	0.1711<br>(0.0039)	£20287<br>(£785)<br>n=221	0.1700<br>(0.0041)	-£1432<br>(£1056)	0.0011<br>(0.0054)	Restrictive<br>dominant<br>(-£1,332,993)

CABG, Coronary artery bypass grafting; eGFR, estimated Glomerular Filtration Rate; ICER, Incremental cost-effectiveness ratio; QALY, Quality-adjusted life-year; SE, Standard error

Notes:

** The interaction term between subgroups for QALYs was statistically significant (p=0.003)*

Appendix References

1. Department of Health. National Schedule of Reference Costs 2012-13. London: Department of Health, 2013.
2. Murphy MF, Murphy GJ, Gill R, et al. National Comparative Audit of Blood Transfusion: 2011 Audit of Blood Transfusion in Adult Cardiac Surgery. 2013. URL: http://hospital.blood.co.uk/media/26859/nca-2011_use_of_blood_in_adult_cardiac_surgery_report.pdf (accessed 30 July 2015).
3. NHS Blood and Transplant. NHS Blood and Transplant Price List 2012-2013. 2012. URL: http://hospital.blood.co.uk/products/ (accessed 28 September 2012).
4. Joint Formulary Committee. British National Formulary. No. 66. London: BMJ Group and Pharmaceutical Press 2013.
5. Davies L, Brown TJ, Haynes S, et al. Cost-effectiveness of cell salvage and alternative methods of minimising perioperative allogeneic blood transfusion: a systematic review and economic model. *Health Technol Assess* 2006;10(44). doi:10.3310/hta10440.
6. Joint Formulary Committee. British National Formulary. No. 47. London: BMJ Group and Pharmaceutical Press 2004.
7. Joint Formulary Committee. British National Formulary. No. 58 London: BMJ Group and Pharmaceutical Press 2009.
8. Department of Health Commercial Medicines Unit. Electronic Marketing Information Tool (eMIT). Drugs and Pharmaceutical Electronic Market Information (eMit). URL: http://cmu.dh.gov.uk/electronic-market-information-tool-emit/ (accessed 10 February 2014).
9. Lord J, Willis S, Eatock J, et al. Economic modelling of diagnostic and treatment pathways in National Institute for Health and Care Excellence clinical guidelines: the Modelling Algorithm Pathways in Guidelines (MAPGuide) project. *Health Technol Assess* 2013;17(58). doi:10.3310/hta17580.
10. Gray AJ, Goodacre S, Newby DE, et al. A multicentre randomised controlled trial of the use of continuous positive airway pressure and non-invasive positive pressure ventilation in the early treatment of patients presenting to the emergency department with severe acute cardiogenic pulmonary oedema: the 3CPO trial. *Health Technol Assess* 2009;13(33). doi:10.3310/hta13330.
11. National Institute for Health and Clinical Excellence. NICE Medical Technology Guidance 8: The VeriQ System for Assessing Graft Flow During Coronary Artery Bypass Graft Surgery. 2011. URL: http://guidance.nice.org.uk/mtg8 (accessed 30 July 2015).
12. National Institute for Health and Clinical Excellence. Nutrition Support in Adults: Oral Nutrition Support, Enteral Tube Feeding and Parenteral Nutrition. Costing Report. Implementing NICE Guidance in England. NICE Clinical Guideline no. 32. 2006. URL: www.nice.org.uk/guidance/cg32/resources/costing-report-194884669 (accessed 10 November 2015).
13. NHS Business Services Authority, NHS Prescription Services. Drug Tariff. The Electronic Drug Tariff. Department of Health; 2014. URL: www.ppa.org.uk/ppa/edt_intro.htm (accessed 21 February 2014).
14. Curtis L. Unit costs of health and social care 2013. Canterbury: Personal Social Services Research Unit, University of Kent. 2013.
15. NHS Direct National Health Service Trust. NHS Direct National Health Service Trust Annual Report and Accounts 2012/13. London: The Stationery Office; 2013. URL: www.gov.uk/government/publications/nhs-direct-national-health-service-trust-annual-report-and-accounts-2012-to-2013 (accessed 30 July 2015).
16. Briggs A, Clark T, Wolstenholme J, et al. Missing... presumed at random: cost-analysis of incomplete data. *Health Econ* 2003;12(5):377-92 doi:10.1002/hec.766.
